# How Infants and Young Children Learn About Food: A Systematic Review

**DOI:** 10.3389/fpsyg.2017.01046

**Published:** 2017-07-25

**Authors:** Manon Mura Paroche, Samantha J. Caton, Carolus M. J. L. Vereijken, Hugo Weenen, Carmel Houston-Price

**Affiliations:** ^1^Danone Nutricia Research Utrecht, Netherlands; ^2^School of Health and Related Research, Section of Public Health, University of Sheffield Sheffield, United Kingdom; ^3^School of Psychology and Clinical Language Sciences, University of Reading Malaysia Iskandar Puteri, Malaysia

**Keywords:** infant, toddler, food preference, learning, eating habits, development

## Abstract

Early childhood is a critical time for establishing food preferences and dietary habits. In order for appropriate advice to be available to parents and healthcare professionals it is essential for researchers to understand the ways in which children learn about foods. This review summarizes the literature relating to the role played by known developmental learning processes in the establishment of early eating behavior, food preferences and general knowledge about food, and identifies gaps in our knowledge that remain to be explored. A systematic literature search identified 48 papers exploring how young children learn about food from the start of complementary feeding to 36 months of age. The majority of the papers focus on evaluative components of children's learning about food, such as their food preferences, liking and acceptance. A smaller number of papers focus on other aspects of what and how children learn about food, such as a food's origins or appropriate eating contexts. The review identified papers relating to four developmental learning processes: (1) *Familiarization* to a food through repeated exposure to its taste, texture or appearance. This was found to be an effective technique for learning about foods, especially for children at the younger end of our age range. (2) *Observational learning* of food choice. Imitation of others' eating behavior was also found to play an important role in the first years of life. (3) *Associative learning* through flavor-nutrient and flavor-flavor learning (FFL). Although the subject of much investigation, conditioning techniques were not found to play a major role in shaping the food preferences of infants in the post-weaning and toddler periods. (4) *Categorization* of foods. The direct effects of the ability to categorize foods have been little studied in this age group. However, the literature suggests that what infants are willing to consume depends on their ability to recognize items on their plate as familiar exemplars of that food type.

## Introduction

The first years of life are marked by tremendous physical and psychological developments, allowing infants to gradually become less helpless and more independent. During this period, infants show rapid advances in their language skills, social awareness, and cognitive capacity for attention and learning (Snow and McGaha, 2003; Goswami, 2008a,b). At the same time, infants undergo significant developments in the eating domain, with the transition from a complete reliance on milk, underpinned by the newborn infant's well-organized sucking reflex, to the omnivore eating behavior of the toddler. The start of this transition is the onset of weaning, when the infant is first introduced to solid foods. Complementary feeding is usually recommended to occur at around 6 months (World Health Organization, 2009; British Dietetic Association, 2016), although some suggest that weaning might begin at any time between 4 and 6 months (e.g., European Society for Pediatric Gastroenterology Hepatology and Nutrition; Agostoni et al., 2008). Regardless of such official guidelines, some parents introduce solid foods even earlier, some as early as 8 weeks of age (Caton et al., 2011). At first, children do not make their own food choices but rely on their caregivers to provide them with appropriate foods. Within 3 or 4 years, the child establishes autonomous feeding behavior, and sets boundaries on the foods they will accept (Hammer, 1992). The “food learning” journey is therefore characterized by a gradual change from total dependence on caregivers prior to weaning to the child becoming an accomplished eater, making independent food choices albeit limited by the context of what is available (Vereijken et al., 2011). The eating behaviors established during this early period track into adolescence and adulthood and, when they are healthy behaviors, may have a positive influence in combatting non-communicable diseases (Skinner et al., 2002; Vereecken et al., 2004; Coulthard et al., 2010).

Importantly, while human infants show similar affective responses toward different taste stimuli across cultures, suggesting a biological underpinning for the foods we are programmed to prefer and avoid (Mennella and Ventura, 2011), infants actually begin life with very few innate taste preferences, and a strong capacity to learn to like new foods (Davis, 1939). The environment—and the family home in particular—play a crucial role in shaping children's eating behaviors (Kral and Faith, 2007). It has been suggested that the introduction of complementary feeding is the most important time for learning about new foods, as it is during this period that the child's senses are suddenly exposed to a variety of new types of stimulation (Lipsitt et al., 1985). Cashdan (1994) found that children younger than 24 months of age were more receptive to new foods than older children, and recommended that parents should introduce new foods at this time. These suggestions support Kolb's (1984) view of the first 3 years of life as a sensitive period for the development of “perception, cognition, behaviors and experiences” in relation to food.

If the period from weaning to around 3 years of age is indeed of major importance for learning about food and developing lifelong preferences, the effective promotion of healthy eating habits in this age group would be facilitated by a better understanding of the mechanisms that support children's learning. A seminal paper by Birch and Anzman (2010) identified three learning processes relevant to children's early learning about food and eating (see also Birch and Doub, 2014). *Familiarization* refers to the positive impact of repeated exposure on liking of the exposed stimulus (Zajonc, 1968). *Associative learning* or *conditioning* occurs when a positive evaluation of a stimulus arises through its association with a second, already-liked stimulus (Birch and Anzman, 2010). *Observational learning* or *social learning* refers to the natural human inclination to observe and imitate the behaviors of others (Bandura, 1977). Birch and Anzman (2010; see also Birch and Doub, 2014) show that these three learning theories each play a role in young children's learning about food. Other work suggests that *categorization* processes—the mental grouping together of stimuli into categories and schemas (Rakison and Oakes, 2003)—also play an important role in children's learning about foods (Nguyen, 2007b). Despite the importance of this area, no systematic review has so far been conducted of the literature relating to the learning processes involved in infants' developing knowledge of food between weaning and 3 years of age. This review aims to fill this gap.

Following Kolb (1984), we consider “learning” to include developments in children's perception, cognition, behavior and experiences in relation to food. Within the target group and age range (human infants between weaning and 36 months), we include all possible aspects of what has to be learned about food. This includes amongst others:

- A food's evaluative status (whether it is liked or disliked, healthy or unhealthy);
- Perhaps most importantly, which foods the child will accept into their diet;
- How foods are recognized based on their physical characteristics (taste, smell, texture and appearance);
- The origins of foods (e.g., whether they are plant or animal products), and how they are prepared;
- The names of food ingredients, preparation processes, utensils, etc.;
- The contexts in which foods are eaten (appropriate times or occasions, quantities and combinations);
- How foods are eaten (including oral-motor skills, regulation of food intake, mealtime etiquette);
- The post-ingestive consequences of consuming foods (whether they are satiating, provide energy, cause nausea, are unsafe or inedible).

In summary, the goal of this systematic review is to provide an overview of the developmental processes that are relevant to how children learn about food. We summarize the relevant empirical evidence in relation to each process (from the start of complementary feeding to 36 months of age), and define the key gaps in the literature that need to be addressed if we are to increase our understanding of early food-related behavior.

## Methodology

**Figure 1:**
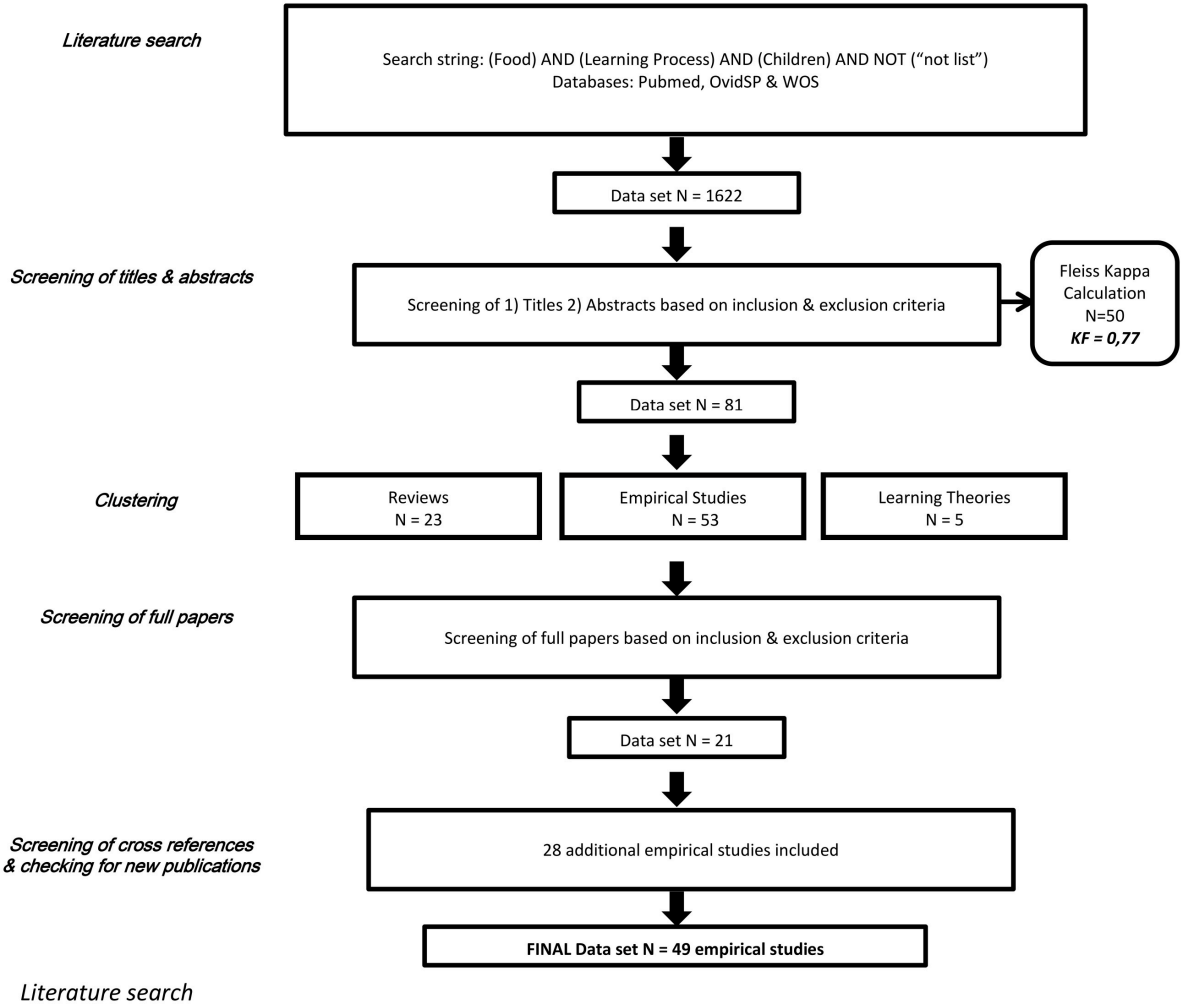
Flow chart of the search and screening methods adopted in the systematic review.

The literature search, screening and clustering methods employed in the systematic review are summarized in Figure 1 and described in more detail below.

### Literature search

The goal of the systematic review was to identify the role played by developmental learning processes in how children learn about food from weaning to 36 months of age. Three groups of search terms were defined: one for “food,” one for “learning process” and one for “children.” For “learning process” the list of search terms included terms relating to all learning theories known to the authors. The list of keywords used as search strings can be seen in Table 1. As a preliminary search generated a large number of irrelevant articles, a “NOT list” of search terms was generated by the authors on the basis of the preliminary search (see Table 1). The search was limited to peer-reviewed articles written in English. The initial literature search was conducted first in February 2012, and then repeated in February 2016, using OvidSP, Pubmed and Web of Science. The initial search resulted in a total of 1622 papers (853 from OvidSP, 811 from Web of Science and 59 from PubMed).

**Table 1:** Search strings used in the literature search.

**Search in Title and Abstract**
Search term 1: FOOD (feed or food or eat^*^ or taste or intake)
Search term 2: (**AND**) LEARNING (habits or socialization or socialization or enculturation or cognit^*^ or social learning or conditioning or imitation or categorization or categorization or programming or schemas or script^*^ or modeling or preference)
Search term 3: (**AND**) CHILDREN (baby or infant or infancy or child^*^ or early life or toddler^*^)
(**NOT**) (alcohol or disorder^*^ or teen^*^ or allerg^*^ or school-age^*^ or sick or ill^*^ or disease^*^ or adipos^*^ or advertis^*^ or TV or television or adolesce^*^ or preterm or supplement or vaccine or autism or dysphagia or defiency or policy or chimpanzee^*^ or birth weight or colonization or rat^*^ or sport or physical activity or cancer or carcino^*^ or cost^*^ or poverty or prenatal or pregnan^*^ or HIV or education or school program or school or education program^*^ or adult^*^ or older or elder or elderly or subject or women or men or gender or blood concentration or plasma concentration or carries or caries or dentifrice or fluor^*^ or disable^*^ or fish^*^ or vitamin D or low income or zinc or copper or nitrate or PCB)
(**AND**) Peer reviewed
(**AND**) English language

### Screening of titles and abstracts

The titles and abstracts of the articles identified by the literature search were screened by hand using the following inclusion and exclusion criteria:

#### Populations

Articles addressing healthy children from weaning to 36 months old were included. Studies with fewer than five participants or involving animals were excluded, as were studies with a clinical or disease focus. Studies of food refusal, picky eating and other non-clinical “problematic” feeding behaviors were included.

#### Focus

Only articles relevant to a learning process in the food domain were included. Articles dealing with the pre-weaning milk-feeding period were excluded, as were studies focusing on the learning shown by parents, rather than children.

#### Type of article

Only articles that were published in English, with named authors, and subject to international peer review were included. Studies focusing on the development of a methodology were excluded, as were conference abstracts and position papers.

Screening involved two steps. The first step involved screening titles; this reduced the set of papers to 366. The second step involved screening of abstracts; this reduced the set of papers to 81.

### Checking the reliability of screening

To check inter-rater reliability, 50 papers were randomly selected from the 366 articles remaining after the first step in the screening process. Four of the authors completed step two for these 50 papers, each making an independent judgment about their inclusion. The Fleiss's Kappa statistic (FK = 0.77) indicated an acceptable level of reliability between their judgments. Disagreements were discussed and a consensus reached in all cases. The remaining papers from step one were then assessed by the first author and a second assessor.

### Clustering

To facilitate the structure of the review, papers that passed screening were clustered. Four clusters were identified, each representing a separate learning process: (1) Familiarization; (2) Observational learning; (3) Associative learning; (4) Categorization. The choice of these clusters was based on a preliminary screening of the selected papers and on the learning processes previously identified as involved in the development of eating habits (Nguyen and Murphy, 2003; Birch and Anzman, 2010).

### Screening of full articles

Two authors were assigned to each of the learning theories. Both authors read all of the articles in their category and excluded any article that was deemed not relevant to how children learn about food. Articles that included participants older than 3 years were not excluded if they also included younger participants. Only reports of empirical studies were included. Screening reduced the set of relevant articles to 20.

### Addition of further articles through cross-referencing and checking for new publications

Further articles were identified through their citation in the papers found in the literature search or because they cited papers found in the search. 17 additional articles were found that satisfied our inclusion/exclusion criteria. A second iteration of the literature search and screening process was conducted in February 2016, following exactly the same process as the first search; this identified 11 newly-published articles that satisfied our inclusion/exclusion criteria and these were added to the set. The final set of articles considered in the review consists of 48 papers (see Tables 2–5).

**Table 2:** Summary table—studies of familiarization through repeated exposure.

**Author(s) and Year**	**Objectives**	**Country and sample**	**Methodology**	**Key findings for food learning**	**QA**	**How children learn**	**What is learned**
Ahern et al., 2013	Examine children's experience with vegetables across three European countries	UK, Denmark and France—234 children of 6–36 months	Survey assessing parental and infant familiarity, frequency of offering and liking of 56 vegetables and preparation techniques for these vegetables	UK children's liking of vegetables was related to frequency of maternal intake and frequency of offering, suggesting learning via modeling and repeated exposure. The authors conclude that increasing variety and frequency of vegetable offering between 6 and 12 months, when children are most receptive, may promote vegetable consumption in children	10	**Observation** of their mother eating a vegetable; **repeated exposure** to taste of vegetable	**Acceptance** of vegetables
Barends et al., 2013	Determine the effects of repeated exposure to either vegetables or fruits on similar food acceptance at the beginning of weaning	Netherlands—101 children from 4 to 6 months old	Infants were assigned to 2 intervention groups: a vegetable group received either green bean or artichoke puree on alternate days, with two other vegetables on the other days; the fruit group received either apple or plum puree on alternate days, with two other fruits on other days, for 18 consecutive days. On day 19, the vegetable groups consumed their first fruit puree and the fruit groups their first vegetable puree	Both vegetable and fruit intake increased significantly in the vegetable group from days 1 and 2 to days 17 and 18. The fruit group consumed no more of the green beans on day 19 than the vegetable group had consumed at the 1st day of exposure. Fruit intake was significantly higher than vegetable intake from the start	10	**Repeated exposure** to the taste of new fruits or vegetables	**Acceptance** of the exposed fruits or vegetables
Beauchamp and Moran, 1984	Examine the relationship between frequency and type of experience with sweet substances and sweet preference at birth and 6 months	USA—63 children from birth to 2 years old	Infants' taste preferences were tested at birth and 6 months with sucrose solutions of different concentrations in the following order: plain water, 0.2, 0.6, 0.6, 0.2 M sucrose. At 2 years of age, children were given a series of 3 tests, separated by at least 7 days, in which their acceptance of sweet tastes was evaluated as at birth and 6 months	At 6 months and 2 years, children who had been regularly fed sugar water consumed more of the sucrose solutions (but not more plain water) than children who had not been fed with sugar water. When tested with sucrose in a fruit-flavored drink, prior exposure to sugar water was unrelated to consumption of the sweetened or unsweetened fruit-flavored drink	8	**Repeated exposure** to a sweet taste food; **formation of schema** that specify a food's usual taste	**Expecting and liking** a specific food to have a sweet taste
Birch et al., 1987	Determine the relative effectiveness of visual and taste exposure on young children's preference for novel foods	USA—43 children from 23 to 69 months	Children received either “look” or “taste” exposures to seven novel fruits. Foods were exposed 5, 10 or 15 times and one remained novel. After exposure, children were assigned to make two judgments of the 21 food pairs based either on looking or tasting, and choosing the one they liked the best	Visual exposure enhanced visual preferences and taste exposure enhanced taste preferences. The visual exposure effect disappeared when taste preference judgments of the same foods were requested. This finding is consistent with a “learned safety” interpretation of exposure effects	10	(a) **repeated exposure** to taste of new fruits (b) **repeated exposure** to visual appearance of new fruits	(a) **liking** of the taste and appearance of the fruits (b) **liking** of the appearance of the fruits
Birch et al., 1998	Establish the number of exposures needed to increase intake of a novel target food and evaluate generalization of liking to other foods	USA—39 children from 4 to 7 months	Infants were repeatedly exposed to 1 target food (one brand of fruit or vegetable) for 10 days at home. Intake of the same food (different brand), similar food (same category), home-prepared food and different food were measured pre- and post-exposure	The greatest change in intake of the target food was observed prior to the start of the exposure phase. Repeated exposure enhanced intake only of the similar food. Post-exposure intake of the target, same and similar foods was significantly greater than intake of the different and home-prepared foods	8	**Repeated exposure** to the taste of a branded fruit or vegetable product	**Acceptance** of the exposed food across brands and acceptance of other similar foods
Blossfeld et al., 2007a	Is consumption of fruit associated with acceptance of sour taste	Ireland—53 children at 6, 12, and 18 months	Fruit intake and acceptance (measured as intake) of 4 drinks with varying degrees of sourness was measured at 6, 12, and 18 months	Children who accepted the drinks with highest sourness had better fruit acceptance at 6 and 18 months	9	**Repeated exposure** to (sour tasting?) fruit increases sour taste and fruit acceptance	**Acceptance** of fruit
Blossfeld et al., 2007b	Identify factors affecting food texture acceptance by infants	USA—70 children aged approx. 12 months	Children were exposed to cooked carrots with 2 different textures: pureed and chopped. Mothers also completed a questionnaire about the child's food habits, age of weaning, pickiness, milk feeding, the child's familiarity with textures and the frequency of exposure to novel foods	At 12 months infants consumed more pureed carrots than chopped carrots. Children with more teeth were more accepting of the chopped texture. Infants' intake of chopped carrots was predicted by previous experiences with different textures. Breast-feeding duration, food responsiveness, dietary variety and willingness to consume new foods were good predictors of intake of chopped carrots	8	**Exposure** to a variety of textures	**Acceptance** of a new complex texture
Bouhlal et al., 2014	Compare effect of repeated exposure and flavor-flavor learning on the acceptance of a non-familiar vegetable	France—151 children 2–3 years	Toddlers were exposed 8x to 1. basic salsify puree 2. same salsify puree with extra salt 3. salsify puree as in 1 with nutmeg	Salsify intake increased from pre- to post-exposure, but no difference between groups was observed on the increase in liking of the target vegetable	11	**Repeated exposure** increases the acceptance of a non-familiar vegetable	**Acceptance** of a non-familiar vegetable
Caton et al., 2013	Compare the effectiveness of different learning strategies	UK—72 children aged between 6 and 38 months. Also Included in Caton et al. (2014)	Children were randomly assigned to one of three conditions (repeated exposure/FFL/FNL) and were offered 10 exposures to a novel vegetable (artichoke). Pre- and post-intervention measures of artichoke puree and a control puree (carrot) intake were taken	Intake of both vegetables increased over time, with a greater increase for artichoke. Artichoke puree increased to the same extent in all three conditions, and this effect persisted for 5 weeks. Five exposures were sufficient to increase intake in the FFL/FNL conditions and 3 exposures in the repeated exposure condition	11	**Repeated exposure** to the taste of a new vegetable; no added value from **associating the** new vegetable with high energy content or sweet flavor	**Acceptance of** the new food
Caton et al., 2014	Compare the effectiveness of different learning strategies	332 children (4–38 months) in UK, Denmark and France	As Caton et al. (2013) but combined with data from similar studies in France and Denmark. The paper focuses on intake of artichoke and analyzing clusters of children	Children in the added energy condition showed the smallest change in intake over time, compared to those in the basic or sweetened artichoke condition. Contrary to expectation the FNL was less effective than RE. Another interesting insight from the paper is that the children could be clustered according to their eating behaviors into learners, plate-clearers, non-eaters and others	11	**Repeated exposure** to the taste of a new vegetable; no added value from **associating** the new vegetable with high energy content or with sweet flavor	**Acceptance** of the new food
Forestell and Mennella, 2007	Evaluate the effects of breastfeeding and dietary experience on acceptance of green vegetables and fruit	USA—45 children aged between 4 and 8 months	Infants were assigned to 2 intervention groups: (1) green beans (GB), (2) GB + peach (GB-P), during an 8-day home exposure regime. Acceptance (intake, records of facial expression, rate of consumption) of green beans was evaluated on days 1 and 11, and acceptance of peach was evaluated on days 2 and 12	Both groups tripled their intake of GB by the end of the exposure period. Children in the GB-P group showed fewer negative facial expressions during test days 11 and 12. Breastfeeding conferred no advantage for acceptance of green beans either before or after exposure	8	(a) **repeated exposure** to the taste of a new vegetable (b) **repeated exposure** to the taste of a fruit-vegetable combination	(a, b) **acceptance** of the vegetable (b) **liking of** the taste of both foods
Gerrish and Mennella, 2001	Evaluate whether the acceptance of novel foods is facilitated by providing a variety of flavors during the weaning period	USA—48 formula-fed children with a mean age of 4.5 months	Children were assigned to 3 intervention groups: (1) pureed carrot (target vegetable), (2) pureed potatoes, (3) variety of vegetables (pureed potatoes, peas and squash), for a 9-day exposure intervention. Infants' acceptance of the target vegetable (carrot) and a novel food (chicken) was evaluated (by intake and videotape) after exposure	Groups 1 and 3 increased their intake of pureed carrot and ate more quickly. Group 3 also increased in acceptance of the novel food. Daily experience with fruits (based on an intake questionnaire) enhanced infants' initial acceptance of carrots	8	(a) **repeated exposure** to the taste of a new vegetable (b) **repeated exposure** to a variety of vegetables	(a,b) **acceptance** of the exposed vegetable (b) **acceptance** of a new food from a different category
Harris and Booth, 1987	Explore the development of salty food preference and its link with previous exposure to dietary sodium	UK—35 children tested at 6 and 12 months	Infants were tested at 6 and 12 months of age for their preference for salt in familiar foods: unsalted and salted cereal at 6 months and unsalted and salted potatoes at 12 months. The relationship between preference for salted food and the infant's dietary experience was examined	At 6 months a positive correlation was found between preference for the salted test food and the amount of experience the child had with high-sodium foods prior to testing. At 12 months this relationship was affected by the order in which food samples were presented and the infant's familiarity with the tested food	7	**Repeated exposure** to salty foods; **formation of schema** that specify a food's usual taste	**Liking** of similar food with added salt
Hausner et al., 2012	Compare the effectiveness of different learning strategies	Denmark—104 children aged between 22 and 38 months. Also Included in (Caton et al., 2014)	Children were assigned to 3 intervention groups (mere exposure/FFL/FNL) and were offered 10 exposures to a novel vegetable (artichoke). Measures of artichoke puree and carrot puree (control vegetable) intakes were taken pre- and post-intervention and at 3- and 6- months follow-up	Children's intake changed by the 5th presentation in the repeated exposure condition and by the 10th exposure in the FFL condition. Mere exposure had the largest impact on intake of unmodified puree both immediately after exposure and at the 6-month follow-up. Children in the FFL group ate more of the sweet puree. In the FNL group, no increase in intake was observed; lower amounts of carrot puree were eaten immediately after the intervention	11	(a) **repeated exposure** to the taste of a new vegetable (b) **association** of new vegetable with sweet flavor	(a) **acceptance of** exposed vegetable (b) **acceptance** of vegetable with added sugar
Heath et al., 2014	Explore the effects of exposure to pictures of liked, disliked and unfamiliar vegetables on children's willingness to look at and taste these	UK—Study 1: 154 children aged between 19 and 26 months. Study 2—68 children aged 20–24 months	Children looked at a picture book about a target vegetable with their parents every day for 14 days, after which they took part in a visual preference test (Study 1) or taste test (Study 2), to measure their interest in looking at or eating the target vegetable vs. a matched control vegetable	Study 1: Children looked longer at pictures of the target food than at pictures of the matched control food. Study 2: Children were more easily persuaded to eat the target food than a matched control vegetable, and consumed more of the target food. In both studies, the strongest exposure effects were seen for initially unfamiliar foods	10	**Repeated exposure** to visual appearance of liked, disliked and unfamiliar vegetables	**Willingness to try** a novel food; **acceptance** of the exposed vegetable
Houston-Price et al., 2009	Explore the effects of exposure to pictures of fruits and vegetables on children's willingness to taste the foods	UK—20 children aged between 21 and 24 months (mean age 23.2 months)	Children received daily exposure to either picture-book A or B (each including 2 fruits and 2 vegetables, 1 of each familiar to children) for 2 weeks. At test, children were offered the exposed and non-exposed foods to eat, and the order in which foods were tasted was recorded	Children displayed a neophobic pattern of behavior toward foods to which they had not been exposed, but not toward exposed foods. Exposure decreased children's willingness to taste familiar vegetables, but increased their willingness to taste unfamiliar fruits	8	**Repeated exposure** to visual appearance of familiar and new fruit and vegetables	**Willingness to try** new fruits; **reluctance to try** familiar vegetable
Lundy et al., 1998	Determine whether food texture preferences differ between infants and toddlers and whether experience with textures influences infants' food preferences	USA—Study 1: 24 children aged between 4 and 12 months. Study 2: 12 toddlers aged from 13 to 22 months	Study (1): Infants' feeding sessions were recorded when they were offered 3 different textures of apple sauce (puree, lumpy and diced). Study (2): Toddlers were assigned to 3 intervention groups: (1) 10 days of exposure to a pureed texture followed by 10 days of exposure to a lumpy texture; (2) 20 days exposure to a lumpy texture; (3) 20 days exposure to a pureed texture	Study (1): Infants displayed more negative expressions, negative head movements and negative body movements when presented with more complex textures. Study (2): Toddlers showed more positive head and body movements and more eagerness for complex textures. Results suggest that progressive introduction to difficult-to-chew textures can facilitate acceptance of more complex textures	8	**Progressive exposure** to more complex textures	**Acceptance** of new complex textures
Maier et al., 2007	Assess changes in acceptance of a disliked vegetable with repeated exposures and the influence of breastfeeding and mothers' eating patterns	Germany—49 children with a mean age of 7 months	Infants were offered a liked and disliked vegetable on alternate days for a 16-day period. Infants' intake and enjoyment (9-point scale) were measured. Mothers completed a questionnaire about breastfeeding practices, food neophobia, eating habits, food consumption and acceptance at 9 months	Intake of both liked and disliked vegetables increased over the 16-day exposure, with no longer significant difference between the two after the 8th exposure. Breast-fed infants ate more of the disliked vegetable on the 1st day. Infants who had mothers “high” in neophobia increased their intake of disliked vegetables more rapidly. By 9 months, 63% of the previously disliked vegetables were rated as liked	9	**Repeated exposure** to the taste of a disliked food	**Acceptance** and **liking** of an initially disliked food
Maier et al., 2008	Measure the effects of breast vs. formula feeding and experience with a variety of vegetables during weaning on new food acceptance	Germany and France—147 children with a mean age of 5.2 months	Children (breast or formula-fed) were assigned to one of 3 intervention groups for a 9-day exposure phase: (1) carrots daily, (2) 3 vegetables on alternating days, (3) 3 vegetables each day. Acceptance of new foods was measured by intake and rated liking of new vegetables on the 12th and 23rd days and meat and fish several weeks later	Intake of carrots did not differ between breast- and formula-fed babies on day 1. Daily vegetable variety was more effective than the number of vegetables fed. Breastfeeding was associated with higher intake of the vegetables, but not meat or fish	9	**Repeated exposure** to a variety of vegetables	**Acceptance** and **liking** of both exposed foods and non-exposed vegetables
Maier-Nöth et al., 2016	Same as Maier et al. (2008) but including follow up till 6 years	Same as Maier et al. (2008)	Same as (Maier et al., 2008) plus follow up by survey + experimental veggie liking assessment at 6 years consisting of rating 6 vegetables on a 7 pt scales and measuring ad lib eating of these 6 vegetables	At 6 years children who had been breast fed and children who had experienced high vegetable variety were more willing to taste vegetables, ate more of new vegetables and liked them more	11	**Repeated exposure** to a variety of vegetables	**Acceptance** and **liking** of both exposed foods and non-exposed vegetables
Mennella et al., 2008	Explore the effects of exposure to a specific food or variety of foods on infants' acceptance of fruit and vegetables	USA—74 children aged between 4 and 9 months	Children were assigned to 2 intervention groups: (1) Pear or variety of fruits; (2) Green beans or variety of vegetables, for 8 days of home exposure, followed by a 2-day test session with pears on 1 day and green beans on the other	Eight days of dietary exposure to pears or a variety of fruits resulted in greater consumption of pears but not of green beans. Eight days of vegetable variety between and within meals led to increased acceptance of green beans, carrots and spinach. Infants exposed to only green beans or a variety of foods between meals only increased their intake of green beans	9	**Repeated exposure** to a variety of fruits or vegetables	**Acceptance of new foods** from the same category
Remy et al., 2013	Compare the effectiveness of repeated exposure, flavor-flavor learning and flavor-nutrient learning in improving vegetable acceptance	France—95 infants aged between 5 and 7 months. Also Included in Caton et al. (2014)	Children were randomly assigned to one of 3 conditions (repeated exposure/FFL/FNL) and were offered 10 exposures to a novel vegetable (artichoke). Pre- and post-intervention measures of liking and intake of artichoke puree (target vegetable) and carrot puree (control vegetable) were collected	Intake of artichoke puree significantly increased in the RE (+63%) and FFL (+39%) groups but not in the FNL group; liking increased only in the RE group (+21%). After exposure, the RE group liked and consumed artichoke as much as they did carrot. Learning of artichoke acceptance was stable up to 3 months post-exposure	10	**Repeated exposure** to the taste of a new vegetable; **association** of a food with a liked taste	**Acceptance of** the new food
Sullivan and Birch, 1994	Examine the effects of dietary experience and milk feeding regimen on acceptance of first vegetables	USA—36 children aged between 4 and 6 months	Infants were assigned to be fed salted or unsalted vegetables at home every day for a 10-day period. Infant intake of the target vegetable and video-recorded responses to this were measured before, during, after the intervention and at a 10-day follow-up. Intake of chicken or tofu was also measured before and after the exposure period	Infants increased in acceptance and liking of the novel vegetable in both forms, regardless of which version they had been exposed to. Breastfed infants increased their intake to a greater extent than formula-fed infants; consumption of breastmilk during exposure and vegetable intake after exposure were positively correlated	9	**Repeated exposure** to a vegetable with added salt	**Acceptance** of vegetable with or without added salt **liking** of the vegetable with or without added salt
de Wild et al., 2013	Investigate the efficacy of flavor-nutrient learning for improving intake of novel vegetables	Netherlands—40 children aged between 2 and 4 years (mean age 36 month)	Children were assigned to 2 intervention groups: group 1 received a high-energy variant of one soup (e.g., HE spinach) and a low energy variant of the other (LE endive); for group 2 the pairing was reversed (HE endive, LE spinach). Children consumed the vegetable soups (endive and spinach) twice a week for 7 weeks	An increase in ad lib intake of both vegetables soups was observed, irrespective of the energy content. Children showed a significant increase in liking of the vegetable soup consistently paired with high energy, supporting FNL	11	(a) **repeated exposure** to the taste of a vegetable soup (b) **association** of vegetable soup with energy content	(a) **acceptance of** the exposed food (b) **liking** of the vegetable paired with high energy content

**Table 3:** Summary table—studies of learning through associative principles.

**Author(s) and Year**	**Objectives**	**Country and sample**	**Methodology**	**Key findings for food learning**	**QA**	**How children learn**	**What is learned**
Bouhlal et al., 2014	Compare effect of repeated exposure and flavor-flavor learning on the acceptance of a non-familiar vegetable	France—151 children 2–3 years	Toddlers were exposed 8x to 1. basic salsify puree 2. same salsify puree with extra salt 3. salsify puree as in 1 with nutmeg	Salsify intake increased from pre- to post-exposure, but no difference between groups was observed on the increase in liking of the target vegetable	11	**Repeated exposure** increases the acceptance of a non-familiar vegetable	**Acceptance** of a non-familiar vegetable
Brown and Harris, 2012a	Examine whether disliked foods can act as contaminants to liked foods during infancy	UK—18 children aged 18–26 months (mean age 22 months)	Children were offered a liked food that was touching a disliked food. Their response to this liked food was compared to the responses of a control group	Children were less likely to eat a liked food touching a disliked food. Disliked foods can act as contaminants, suggesting that they may be perceived as disgusting by children	7	**Association** of a liked food with a disliked food	**Rejection** of the liked food
Caton et al., 2013	Compare the effectiveness of different learning strategies	UK—72 children aged between 6 and 38 months	Children were randomly assigned to one of three conditions (repeated exposure/FFL/FNL) and were offered 10 exposures to a novel vegetable (artichoke). Pre- and post-intervention measures of artichoke puree and a control puree (carrot) intake were taken	Intake of both vegetables increased over time, with a greater increase for artichoke. Artichoke puree increased to the same extent in all three conditions, and this effect persisted for 5 weeks. Five exposures were sufficient to increase intake in the FFL/FNL conditions and 3 exposures in the repeated exposure condition	11	**Repeated exposure** to the taste of a new vegetable; no added value from **associating the** new vegetable with high energy content or sweet flavor	**Acceptance of** the new food
Caton et al., 2014	Compare the effectiveness of different learning strategies	332 children (4–38 months) in UK, Denmark and France	As Caton et al., 2013 but combined with data from similar studies in France and Denmark. The paper focuses on intake of artichoke and analyzing clusters of children	Children in the added energy condition showed the smallest change in intake over time, compared to those in the basic or sweetened artichoke condition. Contrary to expectation the FNL was less effective than RE. Another interesting insight from the paper is that the children could be clustered according to their eating behaviors into learners, plate-clearers, non-eaters and others	11	**Repeated exposure** to the taste of a new vegetable; no added value from associating the new vegetable with high energy content or with sweet flavor	**Acceptance** of the new food
Coyle et al., 2000	Evaluate whether pavlovian conditioning methods can be used to increase ingestion of non-preferred solutions	USA—24 formula-fed children aged between 4 and 7 months	During a 3-day olfactory conditioning period, parents placed a scented disk on the rim of their infants' formula bottle at every feeding. Infants' responses to water were tested when their water bottles were scented with: training odor, novel odor or no odor during 3 consecutive trials	Infants sucked more frequently and consumed significantly more water when tested with the training odor than when tested with no odor	7	**Association** of a new food with a flavor previously paired with a liked food	**Acceptance** of the new food
Forestell and Mennella, 2007	Evaluate the effects of breastfeeding and dietary experience on acceptance of green vegetables and fruit	USA—45 children aged between 4 and 8 months	Infants were assigned to 2 intervention groups: (1) green beans (GB), (2) GB + peach (GB-P), during an 8-day home exposure regime. Acceptance (intake, records of facial expression, rate of consumption) of green beans was evaluated on days 1 and 11, and acceptance of peach was evaluated on days 2 and 12	Both groups tripled their intake of GB by the end of the exposure period. Children in the GB-P group showed fewer negative facial expressions during test days 11 and 12. Breastfeeding conferred no advantage for acceptance of green beans either before or after exposure	8	(a) **repeated exposure** to the taste of a new vegetable (b) **repeated exposure** to the taste of a fruit-vegetable combination	(a, b) **acceptance** of the vegetable (b) **liking of** the taste of both foods
Gregory et al., 2010	Explore the association between feeding practices and children's eating behavior	Australia—156 children aged between 2 and 4 years (mean age 3.3 years)	Mothers completed questionnaires about maternal feeding practices, child eating behavior and reported their child's height and weight. The questionnaire was repeated 12 months later	Modeling of healthy eating predicted lower child food fussiness and higher interest in food 1 year later, and pressure to eat predicted lower child interest in food. Restriction did not predict changes in child eating behavior. Maternal feeding practices did not prospectively predict child food responsiveness or child BMI	10	(a) **observation** of adult model eating healthy (b) **association** of foods/eating with parental pressure to eat	(a) enjoyment of eating and **willingness to try** new foods (these are aspects of fussy eating) (b) lower interest in food
Hausner et al., 2012	Compare the effectiveness of different learning strategies	Denmark—104 children aged between 22 and 38 months	Children were assigned to 3 intervention groups (mere exposure / FFL/ FNL) and were offered 10 exposures to a novel vegetable (artichoke). Measures of artichoke puree and carrot puree (control vegetable) intakes were taken pre- and post-intervention and at 3- and 6- months follow-up	Children's intake changed by the 5th presentation in the repeated exposure condition and by the 10th exposure in the FFL condition. Mere exposure had the largest impact on intake of unmodified puree both immediately after exposure and at the 6-month follow-up. Children in the FFL group ate more of the sweet puree. In the FNL group, no increase in intake was observed; lower amounts of carrot puree were eaten immediately after the intervention	11	(a) **repeated exposure** to the taste of a new vegetable (b) **association** of new vegetable with sweet flavor	(a) **acceptance of** exposed vegetable (b) **acceptance** of vegetable with added sugar
Johnson et al., 1991	Assess the conditioning effect of an energy-dense food and the capacity of the child to adjust his/her intake accordingly	USA—20 children aged between 2 and 5 years	Children consumed a fixed quantity of novel-flavored yogurts that were high or low in fat every day for 8 days	Children increased their preference for the flavor paired with the energy-dense yogurt	7	**Association** of new flavor with energy content	**Liking** of the flavor paired with high energy content
Remy et al., 2013	Compare the effectiveness of repeated exposure, flavor-flavor learning and flavor-nutrient learning in improving vegetable acceptance	France—95 infants aged between 5 and 7 months	Children were randomly assigned to one of 3 conditions (repeated exposure/FFL/FNL) and were offered 10 exposures to a novel vegetable (artichoke). Pre- and post-intervention measures of liking and intake of artichoke puree (target vegetable) and carrot puree (control vegetable) were collected	Intake of artichoke puree significantly increased in the RE (+63%) and FFL (+39%) groups but not in the FNL group; liking increased only in the RE group (+21%). After exposure, the RE group liked and consumed artichoke as much as they did carrot. Learning of artichoke acceptance was stable up to 3 months post-exposure	10	**Repeated exposure** to the taste of a new vegetable **association** of a food with a liked taste	**Acceptance of** the new food
Vereecken et al., 2004	Examine differences in mothers' food parenting practices by educational level and their relationship with preschoolers' food consumption	Belgium—316 mother-child pairs, children aged between 2.5 and 7 years (mean age 4.7 years)	Mothers completed a questionnaire covering frequency of food consumption, parenting practices and mothers' educational level. Multiple logistic regression analysis was used to identify variables that predict the consumption of fruits/vegetables/sweets/soft drinks	Mothers' own consumption was an independent predictor of intake of the 4 selected foods; verbal praise predicted children's vegetable consumption; permissiveness predicted regular consumption of soft drinks and sweets; use of food as a reward predicted regular sweet consumption. Differences in children's food consumption by mothers' educational level were completely explained by mother's consumption and other food parenting practices for fruit and vegetables but not for soft drinks	9	(a) **observation** of mother as eating model for specific foods (b) **association** of vegetable consumption with verbal praise	(a) **acceptance of** foods eaten by mother (b) **acceptance** of foods that lead to praise
de Wild et al., 2013	Investigate the efficacy of flavor-nutrient learning for improving intake of novel vegetables	Netherlands—40 children aged between 2 and 4 years (mean age 36 month)	Children were assigned to 2 intervention groups: group 1 received a high-energy variant of one soup (e.g., HE spinach) and a low energy variant of the other (LE endive); for group 2 the pairing was reversed (HE endive, LE spinach). Children consumed the vegetable soups (endive and spinach) twice a week for 7 weeks	An increase in ad lib intake of both vegetables soups was observed, irrespective of the energy content. Children showed a significant increase in liking of the vegetable soup consistently paired with high energy, supporting FNL	11	(a) **repeated exposure** to the taste of a vegetable soup (b) **association** of vegetable soup with energy content	(a) **acceptance of** the exposed food (b) **liking** of the vegetable paired with high energy content

**Table 4:** Summary table—studies of learning through observation.

**Author(s) and Year**	**Objectives**	**Country and sample**	**Methodology**	**Key findings for food learning**	**QA**	**How children learn**	**What is learned**
Addessi et al., 2005	Determine whether the effect of modeling on the acceptance of a novel food is food specific	USA—27 children from 2 to 5 years (mean age 3.9 years)	Children were assigned to one of 3 intervention groups: Presence (a model was present but not eating the food), Different food (model and child ate different foods), Same food (model and child ate the same foods)	Children in the “same food” condition ate more of the novel food than those in the “presence” and “different food” conditions. Children's ages (below or above 45 months), early feeding practices and classroom membership did not affect food acceptance	7	**Observation** of an adult model eating a new food	**Acceptance** of the same food
Ahern et al., 2013	Examine children's experience with vegetables across three European countries	UK, Denmark and France—234 children of 6–36 months	Survey assessing parental and infant familiarity, frequency of offering and liking of 56 vegetables and preparation techniques for these vegetables	UK children's liking of vegetables was related to frequency of maternal intake and frequency of offering, suggesting learning via modeling and repeated exposure. The authors conclude that increasing variety and frequency of vegetable offering between 6 and 12 months, when children are most receptive, may promote vegetable consumption in children	10	**Observation** of their mother eating a vegetable **repeated exposure** to taste of vegetable	**Acceptance** of vegetables
Ashman et al., 2014	Does fruit and vegetable consumption affect fruit and vegetable consumption of off-spring?	Australia—52 pregnant women and their children till 2–3 years	Dietary intake was measured based on food frequency questionnaires, followed by correlation and mediation analysis	Effect of maternal diet during pregnancy on fruit and vegetable intake by their children was mediated through matermal post-natal diet. This supports the role of the mother's current diet on the diet quality of their children at 2–3 years and supports literature that shows that mothers can act as role models for their children by consuming a wide variety of nutritious foods	10	**Observation** of mothers consuming nutritious foods	**Acceptance** of nutritious foods esp. fruit and vegetables
Birch, 1980	Investigate the influence of peer models' food selection and eating behaviors on preschoolers' food preferences	USA—39 children from 2 to 4 years (mean age 3.1 years)	A (target) child who preferred vegetable A to B was seated with 3 or 4 peers with opposite preference patterns. Children were served their preferred and non-preferred vegetable pairs at lunch and asked to choose one. On day 1 the target child chose first, while on days 2, 3, and 4 peers chose first	70% of the children showed a shift from choosing their preferred food on day 1 to choosing their non-preferred food by day 4. Consumption data corroborated these results. In the post-intervention test fewer than half of the peers changed their preferred foods. Younger children were more affected by peer modeling than older children	9	**Observation** of a child role model eating a non-preferred food	**Liking** and **acceptance** of previously disliked food
Edelson et al., 2016	Evaluate how parents' prompts to eat fruits and vegetables are related to children's intake of these foods	USA—60 families with toddlers of 12–36 months	Food diary and video to record child and parent behavior; after 1 week, video recording when child was given a novel fruit or vegetable; after 3 months three 24 h dietary recalls	The most immediately successful prompt for regular meals across food types was modeling. There was a trend for using another food as a reward to work less well than a neutral prompt for encouraging children to try a novel fruit or vegetable	11	**Observation** of an adult model eating a fruit or vegetable	**Acceptance** of fruits and vegetables
Gregory et al., 2010	Explore the association between feeding practices and children's eating behavior	Australia—156 children aged between 2 and 4 years (mean age 3.3 years)	Mothers completed questionnaires about maternal feeding practices, child eating behavior and reported their child's height and weight. The questionnaire was repeated 12 months later	Modeling of healthy eating predicted lower child food fussiness and higher interest in food 1 year later, and pressure to eat predicted lower child interest in food. Restriction did not predict changes in child eating behavior. Maternal feeding practices did not prospectively predict child food responsiveness or child BMI	10	(a) **observation** of adult model eating healthy (b) **association** of foods/eating with parental pressure to eat	(a) enjoyment of eating and **willingness to try** new foods (these are the aspects of fussy eating) (b) lower interest in food
Hamlin and Wynn, 2012	Investigate whether a source's previous pro/antisocial behavior influences imitation of food preferences	USA—48 infants aged 15–16 months	Infants were assigned to one of three conditions (prosocial, novel or antisocial), and exposed to a puppet show where either pro- or anti-social behavior was displayed. During the show the same puppet (or a novel puppet) revealed their favorite food. Following the show infants took part in a food preference test	When presented with a puppet who had demonstrated novel or prosocial behavior, infants chose the food for which the puppet expressed a preference. This effect was not observed for puppets who demonstrated antisocial behavior	11	Selective **observational learning**, based on an infant's evaluation of the individual's behavior	**Acceptance** of a food liked by models demonstrating prosocial or novel behavior
Harper and Sanders, 1975	Determine the impact of adults offering and/or modeling eating of unfamiliar foods to children in the home	USA—80 children aged between 14 and 48 months	Children were assigned to 3 intervention groups: (1) “offer-only condition,” (2) “adult-also-eats condition,” (3) “male/female visitor offer-only condition.” Children were offered 2 new foods at home	Children accepted the food item offered more often when adults were also eating, especially girls. Foods were more often accepted more when presented by the mother than by a visitor, especially by children at the younger end of the age range	8	**Observation** of an adult model (especially the mother) eating a novel food	**Willingness to try** a novel food
Lumeng and Hillman, 2007	Investigate the effect of group size on food intake	USA—54 children aged between 2.5 and 6.5 years	Children took part in two conditions; small group (*n* = 3) and large groups (*n* = 9. Food intake (g) and duration of snack session were recorded	Children consumed approx. 30% more food when eating in a large group compared to a small group if the snack duration was longer than 11.4 min. No group differences in intake were observed when snack duration was shorter than this	10	**Social facilitation** of eating when among larger groups	Greater **acceptance** of snack foods
McGowan et al., 2012	Identify environmental and personal factors predicting intake of core and non-core foods by young children	UK—434 pre-school children (2–5 years)	Self-report survey among caregivers and multiple regression analysis	Only parental intake was important across all types of foods (fruits, vegetables and non-core foods), whereas feeding styles, children's preferences and availability were relevant for selected foods only	11	**Observation** of a parent eating	**Acceptance** of foods eaten by the parent(s)
Morton et al., 1996	Evaluate the eating habits of children between 2 and 3 years of age	Australia—27 children aged between 2 and 3 years	Mothers answered a questionnaire consisting of 32 open-ended questions and a 24-h diet recall for their child	Most mothers reported that their 2-year-old children ate the same foods as the rest of the household. Children were encouraged to eat the foods that were offered to them by seeing parents or siblings as role models. Mothers reported that their children would readily imitate the example of their older siblings	3	**Observation** of an adult or sibling eating	**Acceptance** of foods eaten by the role model
Pliner and Pelchat, 1986	Evaluate the similarities in food preferences between children, their siblings and parents	Canada—55 families with a child aged between 24 and 83 months	Survey of food preferences of the target child, mother, father and sibling. The likes/dislikes of the target children were cross-tabulated with those of their family and Φ-statistics were computed for the child-mother, child-father and child-sibling pairs as measures of similarity in food preferences	Children's food preferences were more similar to those of members of their own family than to those of members of pseudo families (families from same subcultural group and with a child in the target age group). Children more closely resembled siblings in their real and pseudo families than mothers or fathers. The target children's preferences resembled those of their (real) siblings most strongly	7	**Observation** of family members as eating models	**Liking** of the foods liked by other family members
Skinner et al., 1998	Determine the concordance between the food preferences of toddlers and their family members	USA—118 children aged between 28 and 36 months	Mothers, fathers and an older sibling completed a questionnaire about 196 foods commonly eaten across the US. Response categories were: never offered, never tasted, likes and eats, dislikes but eats, likes but does not eat, and dislikes and does not eat	The concordance between mother/child pairs for liked foods was high (78%), while for disliked foods it was very low (4%). Overall, concordance between child/mother, child/father and child/sibling were very high, with no family member more influential than another. 25% of the food items were liked and eaten by at least 85% of children	9	**Observation** of family members as eating models	**Liking** of the foods that are liked by other family members
Skinner et al., 2002	Explore changes in children's food preferences with age and identify influences on these	USA—70 child/mother pairs monitored from 2 to 8 years of age	Mothers completed the Food Preference Questionnaire for children when they were 2 or 3 (T1), 4 (T2) and 8 (T3) years of age, and for themselves at T1 and T3. Changes in food preferences were explored	Mothers reported that, on average, children liked 60% of the 196 foods at T1. This number increased by a non-significant 3.7% during the 5.7 years of the study. The number of foods liked at 4 years of age was the strongest predictor of the foods liked at 8 years and the child's food neophobia score. Mothers tended not to offer their child foods they disliked themselves	11	**Observation** of the mother as eating model for specific foods	**Liking of** the same foods as the mother
Vereecken et al., 2004	Examine differences in mothers' food parenting practices by educational level and their relationship with preschoolers' food consumption	Belgium—316 mother-child pairs, children aged between 2.5 and 7 years (mean age 4.7 years)	Mothers completed a questionnaire covering frequency of food consumption, parenting practices and mothers' educational level. Multiple logistic regression analysis was used to identify variables that predict the consumption of fruits/vegetables/sweets/soft drinks	Mothers' own consumption was an independent predictor of intake of the 4 selected foods; verbal praise predicted children's vegetable consumption; permissiveness predicted regular consumption of soft drinks and sweets; use of food as a reward predicted regular sweet consumption. Differences in children's food consumption by mothers' educational level were completely explained by mother's consumption and other food parenting practices for fruit and vegetables but not for soft drinks	9	(a) **observation** of mother as eating model for specific foods (b) **association** of vegetable consumption with verbal praise	(a) **acceptance of** foods eaten by mother (b) **acceptance** of foods that lead to praise
Wardle et al., 2005	Investigate the association between parental control, child food neophobia and child fruit/vegetable consumption	England—564 children aged between 24 and 72 months (mean age of 45.2 months)	Parents completed a questionnaire with items assessing parents' and children's fruit and vegetable intake, the Parental Control Index, and the Child Food Neophobia Scale	Child fruit and vegetable consumption was positively correlated with parental fruit and vegetable consumption and negatively correlated with child neophobia. No relationship was found with parental control when parental intake and neophobia were controlled for	11	(a) **observation** of parents as models for eating specific foods	(a) **liking** of same foods as parents
Wertz and Wynn, 2014	To examine whether infants identify plants and artifacts as a food source after seeing an adult place these in their mouth	USA—Exp 1: 32 infants aged 18 months. Exp 2: 16 infants aged 18–19 months. Exp 3: 16 infants aged 18 months. Exp 4: 32 infants aged 6 months	Exp 1: infants observed experimenters place a dried fruit attached to a plant or an artifact either in their mouth (food-relevant action) or behind their ear (food-irrelevant action). Fruits were then placed on a tray and infants were asked “which foods can you eat?” in the in-mouth condition or “which foods can you use?” in the behind-ear condition. Exp 2: As in Exp 1 but infants were exposed to in-mouth actions of a fruit from a plant or a more familiar artifact. Exp 3: Infants were exposed only to fruits hanging from a plant or from an artifact. Exp 4: In a violation of expectation paradigm, infants were exposed to the in-mouth action for fruits attached to a plant vs. an artifact	After observing an adult place a food from a plant or artifact in their mouth, 6- to 18-month-old infants are more likely to identify the plant as a food source than the artifact	10	(a) **observational** learning of food sources is selective (b) learning about food sources depends on **categorization** of source as edible	**Acceptance** of item as edible

**Table 5:** Summary table—studies of learning through categorization.

**Author(s) and Year**	**Objectives**	**Country and sample**	**Methodology**	**Key findings for food learning**	**QA**	**How children learn**	**What is learned**
Beauchamp and Moran, 1984	Examine the relationship between frequency and type of experience with sweet substances and sweet preference at birth and 6 months	USA—63 children from birth to 2 years old	Infants' taste preferences were tested at birth and 6 months with sucrose solutions of different concentrations in the following order: plain water, 0.2, 0.6, 0.6, 0.2 M sucrose. At 2 years of age, children were given a series of 3 tests, separated by at least 7 days, in which their acceptance of sweet tastes was evaluated as at birth and 6 months	At 6 months and 2 years, children who had been regularly fed sugar water consumed more of the sucrose solutions (but not more plain water) than children who had not been fed with sugar water. When tested with sucrose in a fruit-flavored drink, prior exposure to sugar water was unrelated to consumption of the sweetened or unsweetened fruit-flavored drink	8	**Repeated exposure** to a sweet taste food; **formation of schema** that specify a food's usual taste	**Expecting and liking** a specific food to have a sweet taste
Brown and Harris, 2012b	Understand how previously liked foods become rejected	UK—Study 1: 312 children aged 6–57 months. Study 2: 89 children aged 12–56 months	Study 1: Parents completed a questionnaire on children's rejection of previously accepted foods. Study 2: Parents completed a questionnaire on children's rejection of previously accepted foods, picky eating and food neophobia	74% (Study 1) and 49% (Study 2) of parents reported at least 1 occurrence of their child rejecting previously accepted foods (PAF), typically vegetables, mixed foods, fruits, brown foods and foods of mixed color. Study 2: Rejection of PAF was related to pickiness and food neophobia. Findings suggest that changes in form and color of PAF may elicit a neophobic response	9	**Formation of schemas** that specify the food's usual form and color	**Rejection** of foods that have an unusual appearance
Cashdan, 1998	Investigate food habits and aversions in children as potentially adaptive behaviors	USA—129 children from birth to 10 years	Parents (mostly mothers) filled in a questionnaire in which they retrospectively described their child's eating behavior at different ages	Some foods refused by children obscured the food's identity (e.g., foods in sauces, mixed foods, pureed items). 52% of toddlers preferred to eat foods separately rather than mixed; only 3% preferred mixtures of foods	6	**Formation of schemas** that specify the food's usual form and color	**Rejection** of foods that have an unusual appearance
Harris and Booth, 1987	Explore the development of salty food preference and its link with previous exposure to dietary sodium	UK—35 children tested at 6 and 12 months	Infants were tested at 6 and 12 months of age for their preference for salt in familiar foods: unsalted and salted cereal at 6 months and unsalted and salted potatoes at 12 months. The relationship between preference for salted food and the infant's dietary experience was examined	At 6 months a positive correlation was found between preference for the salted test food and the amount of experience the child had with high-sodium foods prior to testing. At 12 months this relationship was affected by the order in which food samples were presented and the infant's familiarity with the tested food	7	**Repeated exposure** to salty foods; **formation of schema** that specify a food's usual taste	**Liking** of similar food with added salt
Macario, 1991	Explore children's knowledge of the predictive validity of color in determining food category membership	USA—12 children aged between 2.7 and 3.5 years	Children were asked to identify which of a pair of (identical except for color) food or non-food exemplars was the appropriate color. Production and comprehension of color labels was also tested	Children discriminated the appropriately colored items at above chance levels, for both foods and non-foods; younger children named colors less well. Preliminary evidence was found for a relationship between color name knowledge and the ability to recognize a food as inappropriately colored	7	**Formation of schemas** that specify the food's usual color	**Usual appearance** of familiar foods
Nguyen, 2007b	Explore children's ability to classify items (including foods) into script and taxonomic categories	USA—14 children aged between 2.2 and 3.0 years (mean age 2.6 years)	Matching task (“which is the same kind of thing?”) with a choice of 2 items to match to target. Targets came from a range of categories, including foods	Children were able to classify and cross-classify foods into taxonomic (e.g., fruits) and script (e.g., breakfast foods) categories	9	**Formation of taxonomic and script categories** that specify the food's type and situations in which it is eaten	Awareness of which foods are appropriate to eat in which combinations or situations
Rozin et al., 1986	Explore when children learn what not to eat, i.e., disgusting, dangerous and inappropriate items, and unacceptable combinations of foods	USA—54 children aged between 16 months and 5 years	Children were offered a series of 33 snacks or dinner time foods, inedible, disgusting or dangerous items, or inappropriate combinations of foods to taste; contact with each food was recorded	Children accepted a large number of foods deemed disgusting, dangerous or inappropriate by adults; acceptance of these decreased with age, especially between 16–29 months and 30–42 months; unusual combinations of foods remained acceptable up to 5 years of age	9	**Formation of schemas** for foods and non-food items	**Rejection** of inappropriate items as non-foods
Shutts et al., 2009	Establish whether infants categorize foods by substance rather than shape, as do adults and older children, and therefore whether food constitutes a core knowledge system	USA—Study 2: 40 children aged 9 months. Study 3: 20 children aged 9 months. Studies 4, 5 and 6: 32 children aged 8 months. Study 7: 16 children aged 8 months	Study (2): Infants' looking times were measured toward a display of 2 food items lying on top of each other; when the top item was grasped, either only that item or both items were lifted together. Study (3): as (2), except that two halves of a single food were shown. Studies (4-7): Infants were habituated to an experimenter tasting a food in a specific form and container; test trials showed the habituated stimulus vs. novel food/container/form combinations	Study (2): Infants showed no preference for two foods moving as one or separately; (3): Infants looked longer when single foods broke in two; (4): Infants discriminated changes in food and container; (5): Infants dishabituated equally to changes in food and container; (6 and 7): Infants dishabituated equally to changes in food's color/texture and shape. Overall, there was no evidence that infants treat foods differently to non-foods at 9 months	11	**Formation of schemas** for foods and non-food items	**Characteristics** of foods that are important to attend to—color and texture, rather than shape or container
Wertz and Wynn, 2014	To examine whether infants identify plants and artifacts as a food source after seeing an adult place these in their mouth	USA—Exp 1: 32 infants aged 18 months. Exp 2: 16 infants aged 18–19 months. Exp 3: 16 infants aged 18 months. Exp 4: 32 infants aged 6 months	Exp 1: infants observed experimenters place a dried fruit attached to a plant or an artifact either in their mouth (food-relevant action) or behind their ear (food-irrelevant action). Fruits were then placed on a tray and infants were asked “which foods can you eat?” in the in-mouth condition or “which foods can you use?” in the behind-ear condition. Exp 2: As in Exp 1 but infants were exposed to in-mouth actions of a fruit from a plant or a more familiar artifact. Exp 3: Infants were exposed only to fruits hanging from a plant or from an artifact. Exp 4: In a violation of expectation paradigm, infants were exposed to the in-mouth action for fruits attached to a plant vs. an artifact	After observing an adult place a food from a plant or artifact in their mouth, 6- to 18-month-old infants are more likely to identify the plant as a food source than the artifact	10	(a) **observational** learning of food sources is selective (b) learning about food sources depends on **categorization** of source as edible	**Acceptance** of item as edible

### Quality assessment

Articles were assessed for quality using assessment criteria adapted from Jackson et al. (2008). Quality criteria were based on whether the article provided a clear description/explanation of: (1) the design; (2) the scientific background and rationale; (3) the hypotheses and objectives; (4) the sample; (5) the data analysis; (6) the findings in relation to the hypotheses and objectives; (7) the provision of attrition/exclusion data, and appropriate handling of missing data; (8) the appropriateness of the procedure; (9) consideration of methodological strengths; (10) consideration of the limits of the study; and (11) the study's relevance for theories of learning about food. As such quality criteria are necessarily subjective we used them only to exclude low-scoring outliers in terms of the total scores awarded. Two authors independently rated each paper and discussed any disagreements until consensus was reached. Quality assessment (QA) scores ranged from 3 to 11 (out of a maximum of 11); 48 papers were awarded scores of 6 or higher, satisfying the majority of the rated criteria. One paper received an outlying score of 3 and was excluded from further consideration. Quality assessment (QA) ratings are provided in Tables 2–5, which list the articles relevant to each of the learning theories of interest.

## Summary of literature

The 48 papers that met the quality assessment criteria were grouped according to the learning process(es) they addressed: 24 papers described studies involving familiarization, 12 explored the role of associative learning, 17 reported studies of observational learning and 9 examined the role played by categorization. This fourteen papers investigated more than one learning process. In the following sections, we introduce each identified learning process, summarize the findings of the papers of relevance to it, and highlight gaps in knowledge remaining to be explored.

### Familiarization through exposure

Familiarization to a stimulus through repeated exposure can increase liking of it. Thus, familiarization with the taste of a previously disliked or unfamiliar food can lead to increased liking and intake. The powerful influence of familiarity begins at the very earliest stages of life, when infants ingest flavors while *in utero* and during milk feeding (Mennella et al., 2009; Nehring et al., 2015), and continues into adulthood (Zajonc, 1968). Here we focus on the effects of exposure to specific tastes or foods from the beginning of weaning to 36 months of age. There are several theoretical perspectives on the mechanism that underpins this effect. According to Zajonc, repeated presentation of a stimulus causes a shift in affect toward it. Thus, familiarized foods take on a more positive valence and are simply liked more. Kalat and Rozin (1973) alternatively offer a “learned safety hypothesis,” according to which repeated exposure teaches us that ingestion of a food is not associated with negative consequences and, thereby, that it is safe to eat.

Twenty-four studies have explored the effects of repeated exposure to food in children between the time of weaning and 36 months. One article (Caton et al., 2014) reports a meta-analysis of 3 separate studies (Hausner et al., 2012; Caton et al., 2013; Remy et al., 2013). Findings originate from the UK, USA, Netherlands, Denmark, France, Ireland and Germany. The mean quality assessment score for these papers was 9.3 (range 7–11), indicating that research conducted in this area is generally of high quality. The 24 studies provided exposure in one of four ways: exposure to specific tastes or foods; exposure to a variety of food types; exposure to a variety of food textures; or exposure to a food's appearance.

#### Exposure to specific tastes or foods

The literature confirms that the tastes infants are exposed to at an early age have long-lasting effects on their liking of specific tastes. Beauchamp and Moran (1984) investigated the effects of early exposure to water containing sugar on later acceptance of sweetened water. Infants who were repeatedly exposed to sugar water at 3 months of age showed increased acceptance of sugar water relative to plain water at 2 years of age compared to those who had never tasted it. Harris and Booth (1987) reported that, by 6 months of age most infants have a preference for salty foods, but the strength of this preference was related to the number of times the child had consumed salty foods during the previous week. Interestingly, 12-month-old infants showed no relationship between their preference for salty foods and their recent consumption of these. By this age infants preferred foods to be salty only if that food type usually contained added salt, suggesting that familiarity with specific food-flavor combinations becomes more important with age. Blossfeld et al. (2007a) also reported that exposure to specific tastes early in life is associated with later taste preferences. While sour tastes are generally rejected by infants and young children (Desor et al., 1975; Steiner, 1977), Blossfeld et al. found that some 18-month-old children accepted sour-tasting solutions, and that these children reportedly had higher intake of fruit post-weaning.

Repeated exposure has also been demonstrated to be effective in encouraging infants to consume more of a target food during the weaning period. Sullivan and Birch (1994) reported increased intake of an unfamiliar vegetable (peas or green beans) by 4- to 6-month-old infants after 10 taste exposures to the vegetable. Forestell and Mennella (2007) confirmed that consumption of green beans by 4- to 8-month-olds tripled after 8 days of exposure, while Birch et al. (1998) demonstrated that a single exposure was sufficient to increase intake in infants of this age. Maier et al. (2007) showed that repeated exposure can also increase infants' intake of an initially disliked food. Eight exposures to a disliked vegetable were sufficient to increase intake in 7-month-olds, an effect that was sustained for at least 9 months in two-thirds of the children. More recently, Remy et al. (2013) found that 10 exposures to a new vegetable during weaning increased infants' intake of the food both in the short term and up to 6 months later.

Several studies have shown repeated exposure to a new food to increase intake in infants and toddlers beyond the weaning period. Bouhlal et al. (2014) found that exposing 2- to 3-year-old children to salsify 10 times increased intake of this vegetable, while de Wild et al. (2013) demonstrated that repeated tasting of an unfamiliar vegetable soup (endive) increased intake compared to baseline in children aged 2–5 years. The effects of repeated exposure can also be long-lasting at this age. Five to ten exposures to the taste of an unfamiliar vegetable have been shown to increase intake at 2 weeks (Caton et al., 2013, 2014) or even 6 months (Hausner et al., 2012) after the intervention. The survey study by Ahern et al. (2013) supports this body of evidence in demonstrating that repeated exposure leads to greater liking of vegetables.

#### Exposure to a variety of foods

Exposing an infant or young child to a variety of foods at a young age is effective in promoting liking and intake of both exposed foods and other new foods. Gerrish and Mennella (2001) found that exposure to a variety of foods enhanced acceptance of an unfamiliar food at weaning. In this study, 5-month-old infants consumed more of the target vegetable when they had previously been exposed to either the target vegetable or to a variety of vegetables other than the target food, compared to infants only exposed to potato. Infants in the “variety” condition also consumed more of an unfamiliar meat dish than infants in other conditions. The extent to which exposure to a variety of tastes promotes acceptance of unfamiliar foods may vary according to the specific foods exposed, however. Barends et al. (2013) recently reported that starting complementary feeding with exposure to a variety of vegetables increases acceptance and consumption of fruit and vegetables, while exposing infants to a variety of fruits at the start of weaning does not increase fruit consumption or vegetable acceptance.

Studies that have explored the consequences of exposing infants to a variety of foods suggest that the schedule used to introduce new foods is important. Maier et al. (2008) found that exposure to three different vegetables on a daily basis was more effective in increasing intake in 6-month-olds than alternating presentations so that the vegetables were each tasted once every 3 days. A follow-up study showed that, at 6 years of age, children who had been exposed to the high-variety intervention were more willing to taste new vegetables and liked and consumed more of these (Maier-Nöth et al., 2016). By manipulating the presentation of vegetables within meals and days, Mennella et al. (2008) demonstrated that both within-meal and between-meal variety enhance 6-month-olds' acceptance of a new (non-exposed) vegetable.

#### Exposure to a variety of textures

Lundy et al. (1998) compared the willingness of infants at the weaning and post-weaning stage (4–12 months) and infants in their second year (13–22 months) to accept foods of different textures. The older infants more readily accepted textured foods than younger infants, attributed to their greater experience with textured foods. Similarly, Blossfeld et al. (2007b) reported that 12-month-old infants with prior experience of carrots in a variety of forms (tastes and textures) consumed more chopped carrots than infants who had not experienced such variety. These studies suggest that acceptance of textured foods may be supported by exposure to foods prepared to have different textures.

#### Repeated visual exposure to foods

Repeated visual exposure has also been demonstrated to be effective in increasing children's liking of target foods. Birch et al. (1987) explored the effectiveness of repeatedly exposing children of 2–5 years to either the appearance or taste of novel fruits. They found that asking toddlers to look at foods repeatedly did not affect children's ratings of how much they liked the food's taste. However, children gave higher ratings when asked how much they liked the appearance of the foods they had looked at. As expected, repeated exposure to a fruit's taste was related to acceptance of the food. The authors concluded that to obtain positive changes in preferences, experience with a food must occur in a relevant modality.

More recently, Houston-Price et al. (2009) exposed infants aged 21–24 months to pictures of fruits and vegetables in a picture book every day for 2 weeks, after which children took part in a taste test. Repeated visual exposure increased children's willingness to taste a previously unfamiliar fruit, compared to a non-exposed unfamiliar fruit, suggesting that looking at foods can positively influence eating behavior. However, the same pattern was not shown for vegetables; looking at familiar vegetables decreased children's willingness to taste these, suggesting that the effects of visual exposure may depend on the food type and the child's prior familiarity with it. A larger follow-up study by Heath et al. (2014) showed that picture-book exposure can enhance toddlers' consumption of vegetables, however. In line with the findings of Houston-Price et al. (2009), the strongest effects were seen for previously unfamiliar vegetables. Weaker positive effects were also seen for familiar (liked and disliked) foods in this study, suggesting that earlier concerns about potential negative effects of visual exposure are unwarranted. This recent work suggests that prior visual exposure in the absence of any opportunities to taste the food can support toddlers in accepting new foods, although further empirical studies that corroborate these findings would be required for strong conclusions to be drawn.

#### Familiarization—summary of results and gaps in the literature

Familiarization via repeated exposure is an important means by which children learn about food during the weaning period and early childhood. Children learn to accept specific tastes through experience, and the effects of early familiarization appear to have long-term effects on consumption. For example, one intervention study has shown effects up to 5.5 years after the exposure was provided at the start of complementary feeding (Maier-Nöth et al., 2016), while an observational study has shown that preferences at 2 years of age correlated with preference at 20 years of age (Nicklaus et al., 2005). Repeated exposure to a variety of foods or food textures can also elicit a willingness to accept further new foods or textures into the child's diet. Finally, visual familiarity with foods may also support infants and toddlers in tasting unfamiliar foods.

The majority of studies yielded by our search involved infants younger than 12 months. While repeated exposure appears to be effective for children up to 36 months of age, further research is needed with children at the older end of our age range. A recent study suggests that repeated exposure becomes less effective as a means of increasing vegetable intake with age, at least within the weaning to 36 month age period; children older than 24 months showed less change in acceptance following exposure than younger infants in this study (Caton et al., 2014). This may be related to the neophobia often shown by children at this age (Dovey et al., 2008); it may be difficult to persuade a child who is very reluctant to taste new foods, and who might even refuse to eat previously-liked foods, to engage in repeated tastings of a new or disliked food. We return to this issue—and to the question of how early exposure leads to long-term changes in children's food preferences—in the Discussion section, when we consider the implications of the literature on all four learning strategies in relation to developmental changes in how children learn about food.

### Associative learning

Infants are born with a liking for sweet tastes and a tendency to reject bitter tastes (Steiner, 1979). They must therefore learn to like foods such as vegetables, which have bitter taste components. Such learning may be supported by associative learning (Brunstrom et al., 2005), an umbrella term for processes that support the formation of associations between two stimuli or between a stimulus and a behavior. In the context of learning about food, associations might be formed in several ways, including: (i) flavor-flavor learning (FFL), in which repeated pairing of a new or disliked food with a familiar and liked taste, such as sweetness, leads to acceptance of the food in the absence of the sweet taste; (ii) flavor-nutrient learning (FNL), in which new foods are paired with high-energy dense ingredients, such as fat or maltodextrin, to enhance post-ingestive satiety signals, exploiting children's natural preference for energy-dense foods; (iii) contamination (association of a new food with a disliked food); or (iv) reward (e.g., association of a food with praise).

Twelve studies have explored the role of associative learning in our target age range. Findings originate from the UK, USA, Australia, France and Denmark. The mean quality assessment score for these papers was 9.4 (range 7–11), indicating that the research in this area was generally of high quality.

Early studies of FFL and FNL suggested that children's liking and intake of target foods was influenced by their association with liked tastes or satiety signals. Johnson et al. (1991) demonstrated a preference for a flavor associated with higher energy density (FNL) in children aged 2–5 years. Coyle et al. (2000) demonstrated that olfactory associative conditioning could be used to increase intake of water in 6-month-old infants (FFL). During three feeds, infants were exposed to a scented disc placed around the top of a bottle containing their regular, liked formula milk. Pre-intervention water consumption was measured without the scented disc, and post-intervention water consumption was measured either without the scented disc, with a disc with the familiar scent, or with a disc with an unfamiliar scent. Post-intervention water intake was greatest when infants were exposed to the familiar scent, suggesting that they had learned to like the scent through its association with milk. These findings suggest that olfactory conditioning might be used to support infants' intake of less palatable drinks when these are first introduced.

However, recent studies comparing the effectiveness of associative learning manipulations (in which exposure to the target occurs in association with a liked flavor or satiety) with exposure alone typically find no advantage to manipulations involving conditioning. Forestell and Mennella (2007) investigated whether 6-month-olds' acceptance of a (bitter) vegetable was enhanced when they were offered a (sweet) fruit purée immediately after each taste of the vegetable. Infants' intake of green beans was no greater among infants who tasted peach purée after the beans than in infants who tasted only the beans, suggesting that repeated exposure to the vegetable was sufficient to increase intake. Four recent investigations have directly compared the effectiveness of FFL, FNL and repeated exposure in infants aged between 6 and 36 months (Hausner et al., 2012; Caton et al., 2013, 2014; Remy et al., 2013). In these studies, infants were offered a taste of an unfamiliar vegetable (artichoke) either in sweetened form (FFL), with added oil to boost the food's energy content (FNL), or without any additive (repeated exposure). The addition of sweetener or oil conferred no advantage over repeated exposure in each case; post-intervention intake of artichoke was actually higher in the repeated exposure condition than in either associative learning condition in all four studies.

These studies suggest that the addition of a sweet taste or energy is not necessary to promote the consumption of vegetables in children aged between 6 and 36 months. Indeed, recent evidence suggests that when children are given ad-libitum access to foods, their natural preference for energy-dense foods does not necessarily lead to increased intake of these. For example, de Wild et al. (2013) found no difference between children's intake of an unfamiliar vegetable soup with or without added oil, even though the toddlers who participated in their study reported stronger liking for the soup with greater energy density. Similarly, Bouhlal et al. (2014) showed that exposing children of 2–3 years to salsify on 10 occasions was sufficient to increase intake of this vegetable, and that adding salt or nutmeg added no benefit. The authors conclude that repeated exposure is the simplest route to increase vegetable intake in toddlers.

Other forms of associative learning, such as the pairing of a food with parental reward or pressure to eat, have been shown to impact on preschoolers' and older children's willingness to consume the food. For example, Vereecken et al. (2004) showed that parental verbal praise is positively related to the vegetable consumption of children aged between 2.5 and 7 years, while Gregory et al. (2010) found that parental pressure to eat was related to lower interest in eating among 2- to 4-year-old children, suggesting the formation of positive and negative associations respectively. Evidence that younger 18- to 26-month-old infants are also influenced by the formation of negative associations comes from a study by Brown and Harris (2012a). When a new food was presented alongside a disliked food, and the two foods touched, the disliked food acted as a “contaminant,” reducing infants' willingness to consume the new food.

#### Associative learning—summary of results and gaps in the literature

In summary, while olfactory associative learning might be useful in helping infants to accept otherwise rejected liquids, such as water, the literature is consistent in demonstrating that conditioning techniques such as FFL or FNL provide no advantage over repeated exposure in shaping the food preferences of infants in the weaning and toddler periods. Hence, repeated exposure is preferred as a way to shape food preferences. There is a dearth of evidence on the role played by other forms of associative learning in the age group of interest, however, although studies with older toddlers and school-aged children indicate that positive and negative associations may be formed with foods. Further research is therefore needed to identify whether infants form positive and negative associations on the basis of the physical and social environments in which foods are offered.

### Observational learning

A third body of research concerns the role played by observational learning. Observational learning (also termed “social learning” or “modeling”) involves the observation and imitation of others' behavior (Bandura, 1977). Infants have a natural tendency to imitate, moderated by the emotional quality of the relationship between the observer and model. Parents are therefore highly influential role models, shaping a range of behaviors in their child, including eating behaviors.

From the start of weaning, infants' mealtimes increasingly take place in a family context. This might affect the child's eating behavior in two key ways. First, eating is “socially facilitated” (Clayton, 1978); if others are eating, we are also more likely to eat, and we are quicker to accept new foods (Visalberghi and Addessi, 2000). Second, family mealtimes expose children to the eating behaviors of parents and siblings, who they may try to imitate. Observing others eating a food increases the likelihood that children will want to taste the same food. Thus, social facilitation and observational learning play an important role in determining children's food preferences and eating behavior; they may also teach the child which foods are safe to eat, how they should be eaten, and how much of them it is appropriate to eat.

The search identified 17 studies investigating the role of observational learning, conducted in the US, UK, Australia, Canada, Denmark, France and Belgium. The mean quality assessment for these articles was 9.2 (range 7–11), demonstrating the generally high quality of the research on this topic. Three themes were identified among these papers: (i) the immediate impact of models on children's eating behavior, and the factors that determine the model's influence; (ii) the longer-term consequences of modeling on the development of food preferences; and (iii) similarities between the food preferences and eating behavior of children and family members.

#### Effect of modeling on immediate food acceptance

Several studies demonstrate that children under 3 years of age are influenced by seeing others eat. Lumeng and Hillman (2007) showed that the mere presence of others induces eating in young children (between 2.5 and 6.5 years), as it does in adults. When children were given an extended period in which to snack, the number of children in the group predicted the amount consumed. Edelson et al. (2016) studied the prompts parents use to encourage their children aged 12–36 months to eat specific foods at mealtimes. The prompts observed included pressure to eat, use of another food or a non-food item as a reward, reasoning with the child, and modeling eating the food; the authors found the most successful prompt across food types to be modeling. Harper and Sanders (1975) and Addessi et al. (2005) both found that intake of a new food was greater when children observed a model eating the food than when the child was simply offered the food to eat. It also seems to be important that the food the model is eating looks similar to that offered to the child. Addessi et al. reported that the 2- to 5-year-old children in their study were more likely to accept a food that was the same color as that eaten by a model.

Other work has explored the ages at which children show observational learning of eating behavior. Harper and Sanders (1975) showed that children as young as 14 months old are more likely to eat an unfamiliar food if they see an adult eating it; girls were more strongly influenced by the adult's eating behavior than boys. However, not all adult models are equally effective; in the same study, children between 14 and 20 months old were more likely to accept a food offered but not eaten by the mother than a food offered but not eaten by a visitor. From 24 months of age, parents (Pliner and Pelchat, 1986) act as effective models. Peer modeling is a particularly strong motivator at 3 years of age, when it can arouse a desire to taste non-preferred foods as well as new foods, and to enhance reported liking of these (Birch, 1980). The same study found that peers had less influence over the food choices of 4-year-olds. However, Addessi et al. (2005) reported that peers had no influence over whether children aged between 24 and 45 months old would accept a food eaten by an adult model, suggesting that adults may still have the greater sway over children at this age.

Two further studies have explored the contextual factors that influence social learning of eating behavior. Hamlin and Wynn (2012) found that 15- to 16-month-old infants showed observational learning of the food preferences of puppets if the puppet had not been seen before or had been seen to display prosocial behavior, but not if the puppet had displayed antisocial behavior. Wertz and Wynn (2014) report a series of studies in which infants of 6 and 18 months were more likely to identify items as foods if the source of the food was a plant, rather than an artefact, and when the item was placed into a model's mouth, rather than behind their ear. These studies demonstrate that observational learning about foods is selective, and dependent on infants' prior experiences of where foods come from, how they are acted upon, and whether the model is desirable to imitate.

#### Longer-term impact of modeling healthy eating

The impact of observational learning on longer-term acceptance of a food has also been investigated. Gregory et al. (2010) found that maternal modeling of healthy eating for 3-year-old children was positively correlated with reports of the child's interest in food and negatively correlated with measures of food fussiness and food responsiveness when children were 4 years of age. Gregory et al. also found that children who were reported to enjoy the experience of shared family mealtimes were more positive about trying new foods in a non-modeling context. Other work has revealed links between maternal modeling of healthy eating and consumption of fruits and vegetables by children aged older than 2 years. Using a multifactorial approach, mothers' consumption of fruit and vegetables was found to be a stronger predictor of children's fruit and vegetable consumption than her educational level (a proxy for socio-economic status) and indices of parental feeding practices, such as permissiveness and use of rewards (Vereecken et al., 2004; Wardle et al., 2005). The survey by Ahern et al. (2013) also supports parental modeling as a determinant of children's liking of vegetables.

#### Similarity between the food preferences of children and family members

Several studies have shown similarities in the food preferences of children aged 24–36 months of age and their family members, and Pliner and Pelchat (1986) found that these relationships remain stable until at least 6 years of age. In a recent survey of 550 families of young children, parents' fruit and vegetable consumption was the strongest predictor of children's fruit and vegetable intake (Wardle and Cooke, 2008); others have similarly reported strong correlations between measures of food intake among family members (e.g., Laskarzewski et al., 1980; but see Faith, 2005, for a review of studies finding weaker correlations). Work by Ashman et al. (2014) found an association between a mother's post-natal diet (but not her diet during pregnancy) and the variety of fruit and vegetables in the child's diet at 2 and 3 years of age.

There is some discrepancy in the literature about which family members' eating preferences are most strongly associated with those of their children. According to Skinner et al. (2002), the mother's food likes and dislikes are most strongly associated; they shape the child's food preferences at 2 years of age and continue to do so until the child is 8 years old. However, Skinner et al. (1998) found strong correspondences between the child's food preferences and those of both parents. Pliner and Pelchat (1986) reported that children's food preferences were more similar to those of their own families than to those of members of pseudo families (families from the same subcultural group with a child in the target age range). This finding was especially pronounced for siblings, and children's food preferences were more similar to those of siblings, both real and pseudo (i.e., other children), than to those of their parents.

McGowan et al. (2012) investigated the environmental and individual predictors of young children's intake of core and non-core foods (foods that do not contribute to the nutritional needs of children). Parental intake was the only factor shown to predict children's intake across all types of foods (fruits, vegetables, and non-core foods), while feeding style, children's preferences and availability were predictors for some food types but not others.

#### Observational learning—summary of results and gaps in the literature

The experimental studies reviewed demonstrate that observational learning has an impact on the eating behavior of children in our age range. However, children are not passive imitators of those around them; they draw on prior experience to select which models to learn from. In the longer term, modeling of healthy eating predicts lower food fussiness and is a stronger predictor of children's fruit and vegetable consumption than parenting style or socio-economic status. In addition, correlational studies show that children's food repertoires are similar to those of their families until at least 8 years of age. However, while similarity between the food preferences of children and the people in their immediate environment may result from observational learning of others' food choices, it may also reflect the availability of the foods eaten by those in the child's immediate environment, and/or the child's exposure to these foods. It is difficult then to disentangle the effects of observational learning from the effects of exposure in studies reporting correlations between the food choices of children and their family members. Indeed, the literature suggests that the sharing of food likes and dislikes by family members is not solely due to observational learning by the child; the cultural context in which the family eats and the availability of foods also play a role, as indicated by the shared preferences of children from similar socio-cultural backgrounds.

In contrast to the effects of familiarization on food preferences, which are evident from the beginning of weaning or even earlier, observational learning from adult models has been shown to occur from 14 months of age, while peer modeling has been shown to be effective from about 2 years of age. Whether the later appearance of observational learning is due to genuine changes in the learning strategies employed in the second year or reflects the dearth of research in younger infants remains to be established. Further experimental work is needed to establish the effect of role models on younger infants' food choices, and to corroborate the preliminary evidence that suggests that children change in their sensitivity to different role models as they grow older.

### Categorization

Humans spontaneously organize objects and events into *categories* in order to make sense of the world (Rakison and Oakes, 2003) and store organized bodies of knowledge about the characteristics, functions and properties of these categories as *schemas* (or, in the case of event schemas, as *scripts*). Schemas allow us to generalize our existing knowledge to new members of a category, enabling us to know how to behave toward these immediately, rather than having to learn about each new stimulus we encounter (Piaget, 1953). Children begin to spontaneously categorize objects in their environment within the first half year of life (Mareschal and Quinn, 2001; Rakison and Oakes, 2003) and use scripts to organize their knowledge of events by the second year (Fivush et al., 1987).

Recent reviews suggest that the categorization of the food domain plays an important role in shaping children's food preferences and eating behavior, particularly their reactions to unfamiliar foods (Pliner, 2008; Aldridge et al., 2009). Foods lend themselves to multiple forms of categorization (Ross and Murphy, 1999). The child must first distinguish between foods and non-foods to make appropriate selections of things to eat (Fallon and Rozin, 1983; Rozin, 1990). The food domain is then organized hierarchically into *taxonomic categories*, such that superordinate categories (e.g., fruits) contain subcategories (e.g., berries, citrus fruits), which in turn comprise further subcategories (e.g., cranberries, tangerines). Foods also belong to *thematic categories* of items that commonly co-occur but share no properties in common (e.g., chips and ketchup), and *script categories* of items that play similar roles in an event schema (e.g., breakfast foods) (Nguyen and Murphy, 2003). Nguyen (2008) proposes that we also group foods into *evaluative categories*, based on an assessment of a food as healthy or unhealthy, good or harmful, delicious or disgusting. A food's evaluative category, alongside its status as familiar or unfamiliar, determines whether it is accepted or avoided. Recent studies have shown that children use taxonomic, script and evaluative categories to represent the food domain from 3 to 4 years of age, and can simultaneously cross-classify foods as belonging to more than one category by age 4 Nguyen and Murphy, 2003; Nguyen, 2007a,b, 2008).

The review identified 7 articles that directly explored children's categorization of the food domain, or how this is reflected in their reactions to new foods, in the period from weaning to 36 months. Two further articles on the effects of familiarization also provide some insight into the role played by categorization. The mean quality assessment score for these articles was 8.3 (range 6–11), suggesting some variability in the quality of these articles. The papers address two main questions: (1) What do young children accept as edible items and, relatedly, why are foods that were previously accepted sometimes rejected at a later stage? (2) How are foods categorized by young children?

#### What do young children accept as food?

Previous work has shown that children progress gradually from an initially broad acceptance of many items as potential foods to a narrower, adult-like awareness of what is edible (Rozin and Fallon, 1980; Fallon et al., 1984). The different ages at which children reject unpleasant, dangerous, disgusting and inappropriate items as foods suggest that the development of the non-food category is an incremental process (Fallon et al., 1984; Rozin et al., 1985; Rozin and Fallon, 1987). Rozin et al. (1986) explored this developmental trend in children aged between 16 and 60 months. The youngest group (aged 16–29 months) readily tasted items considered dangerous, disgusting, inappropriate or unacceptable combinations by adults, and acceptance of inappropriate items and unacceptable combinations remained surprisingly high in children older than 30 months. Rozin et al. (1986) suggest that such behaviors reflect children's ignorance of the properties of non-food items that render these inedible; the authors also raise the possibility that young children lack an adult-like categorical distinction between foods and non-foods.

Two studies have investigated the factors that underlie young children's rejection of foods. Cashdan (1998; see also Cashdan, 1994) asked parents to retrospectively complete a questionnaire about their child's food receptivity during early childhood. Rejected foods tended to be those that obscured the identity of component ingredients (e.g., sauce-covered or mixed foods). A subgroup of parents was asked whether their child preferred mixed or individual foods during toddlerhood; parents reported a clear preference for non-mixed foods. Brown and Harris (2012b) investigated the frequency with which children aged between 6 months and 4.5 years reject previously-accepted foods. The majority of parents reported that their child had rejected at least one food they had previously enjoyed eating. The most frequently rejected foods were vegetables and food combinations, and foods that were brown or multi-colored. The authors suggest that such foods may be difficult for children to recognize or classify as a specific food; while the child might have enjoyed a food in the past, changes in its visual appearance might lead it to be rejected as a new or unfamiliar food. Together, the studies by Cashdan (1998) and Brown and Harris (2012a,b) suggest that children prefer foods they are able to recognize as belonging to a particular food type.

Unfamiliar combinations of flavors may also hinder acceptance. Beauchamp and Moran (1984) found that, while prior exposure to sugar water increased infants' consumption of sugared water, it had no impact on intake of sweetened fruit-flavored drinks. Harris and Booth (1987) also reported that 12-month-old infants preferred foods to be salty only when that food type usually contained added salt. Thus, both studies suggest that certain tastes are accepted only in recognized combinations.

#### Categorization of the food domain

Recent work suggests that 2-year-old children categorize foods and non-foods in a similar way to adults. Nguyen (2007b) showed that children of this age are able to cross-classify food and non-food objects on the basis of the item's taxonomic or script category. When asked to select which of two pictures was “the same kind of thing” as a third, target picture, items from the target's taxonomic or script category were selected at above chance levels. Although responses toward food and non-food stimuli were not analyzed separately, the results are suggestive that the food domain may be organized in a coherent manner by 2 years of age.

Some have suggested that infants' knowledge of the food domain might be supported by a “core knowledge” system, which directs our attention to the relevant characteristics of items to help us distinguish category members from non-members (Spelke and Kinzler, 2007). Shutts et al. (2009) explored whether, like adults, older children and monkeys, 8- and 9-month-old infants categorize food and non-food objects by substance (conveyed by color, texture and smell) or by shape (the primary factor in categorization of non-foods). A series of rigorously-controlled looking-time studies found no evidence that infants respond differently to food and non-food objects. Shutts et al. concluded that, rather than possessing innate core knowledge about food, children must learn the relevance of color and texture to food categorization through their experiences in later infancy.

By the late preschool years, however, children base their inferences about foods and non-foods on relevant properties, such as color, texture and smell (Macario, 1991; Lavin and Hall, 2001). For example, when Macario (1991) introduced a novel object as a toy and asked, “Which one is like this one to play with?” 3- and 4-year-old children generalized the item's properties to an object that matched in shape. When the same object was introduced as a food, and children were asked “Which one tastes like this one?” an object of the same color was selected. In a second study, 2- and 3-year-old children matched foods to their appropriate colors, suggesting that color is a relevant property for categorizing foods by 2 years of age.

#### Categorization—summary of results and gaps in the literature

During the first year of life, infants do not distinguish foods from non-foods in either the physical characteristics ascribed to food vs. non-food objects or the restrictions they place on what is considered edible. Children's willingness to consume inappropriate items shows that confusion over membership of the food domain continues well beyond toddlerhood. This is surprising considering that toddlers are able to classify foods according to their taxonomic or script categories by 2 years of age. Children's acceptance of non-food items as foods may therefore reflect their lack of experience with these items. Little is known about how children come to categorize foods in this period except that, by 2 years of age, a food's color is considered an invariant aspect of its identity. The ability to recognize a food by its shape and color is important to toddlers, who prefer foods to be presented separately rather than combined with other foods or hidden in sauce, and who reject foods whose appearance differs to a familiar preparation.

Many questions remain about the structure of children's food categories and how these relate to their preferences. Research is needed to establish how children develop adult-like food categories, and how these are shaped by the child's sensory experiences of foods and/or the behavior of people around them. Little is known about the role played by the names used for food categories and characteristics (i.e., food labels), although recent work has shown that the ability to label a food's flavor is related to children's memory of the flavor (Lumeng et al., 2005), suggesting that knowing the name of a food might impact on its familiarity, and hence on liking. Another remaining question concerns how young children represent foods with conflicting evaluative categories (e.g., delicious but fattening, or disgusting but nutritious). Answering this question would further our understanding of the factors underpinning children's food preferences (Nguyen, 2008).

## Discussion

This article is the first systematic review of the literature on how children learn about food between weaning and 3 years of age. The review clarifies the role played by known learning processes—familiarization, associative learning, observational learning and categorization—in the development of children's understanding of food.

Although attention to the development of eating habits is increasing among researchers, the number of empirical articles relevant to this topic in this age range up to 2016 is only 48. The literature is therefore surprisingly limited in its scope. The large majority of the studies identified was conducted in USA or Europe, with a small number of studies from Australia and none from other parts of the world. As obesity is increasing in many parts of the globe, investigations into children's learning about foods in other regions and cultures are needed. A further limitation of the literature to date is its almost exclusive focus on children's consumption of fruit and vegetables. Other wholesome foods, such as fish and whole-grain cereals, are also under-consumed, but no study involving children less than 3 years has used these as target foods. Perhaps most importantly, most studies have established only short-term effects of interventions on children's knowledge or behaviors toward foods, when longer-term influences are of primary importance. We also acknowledge that our strict inclusion/exclusion criteria may have impacted on the pool of studies that were summarized, and therefore on the conclusions that were drawn. For example, in an effort to focus the review on the learning mechanisms employed by children outside the context of adverse environmental or biological conditions, our search terms excluded studies of food-related behavior in low income groups and atypical populations; we found that studies of food-related learning in “low income” groups often highlight issues of food insufficiency (“food insecurity”), which we considered beyond the remit of the review. It is possible, therefore, that the criteria adopted limit the generalizability of the conclusions to certain sub-groups of children. At the same time, around a third of the studies summarized in the review involved a participant sample that exceeded the upper limit of our target age range. In several of these articles no distinction was made between the behaviors of children younger and older than 36 months, which may have resulted in the inadvertent drawing of conclusions that are inappropriate for our target age group.

The literature search revealed an imbalance in the size of the literature exploring different forms of learning. The majority of studies have investigated the effects of familiarization (*N* = 23); these provide a robust demonstration of the role of repeated exposure. From the start of the weaning period, repeated exposure to the taste of foods helps infants accept these into their diets (Birch et al., 1987 and 20 other papers, see Table 2). At the same age, regularly offering infants a variety of foods enhances their willingness to try other new foods, facilitating the acceptance of a wider variety of foods into the diet (Gerrish and Mennella, 2001 and 4 other papers, see Table 2). By the second year, exposure to a variety of textures enhances acceptance of the more complex textures that are typical of wholesome foods (Blossfeld et al., 2007b). Repeated visual exposure to unfamiliar foods in children of 20 months and older (Houston-Price et al., 2009; Heath et al., 2014) can also support acceptance of these foods.

Fewer papers report findings relating to observational learning (17), associative learning (12) and categorization (9) in infancy, and there remain significant gaps in our understanding of the role of these processes. Within the associative learning domain, there is a paucity of literature on the effects of associations formed with the physical and social environment in which infants eat. Though beyond the scope of this review, studies concerning the impact of parental feeding style and parental feeding practices such as expressed through parental praise, encouragement and pressure to eat, may extend our understanding of how infants and toddlers learn about food. Similarly, how do factors associated with the eating environment (such as the atmosphere at family mealtimes or the presentation of liked and disliked foods together on a plate) influence children's willingness to eat?

Studies of conditioning forms of associative learning have shown that the addition of a sweet taste (flavor-flavor learning, FFL) or energy (flavor-nutrient learning, FNL) confers no advantage over repeated exposure in increasing consumption of the food in the weaning or toddler stages (Hausner et al., 2012; Caton et al., 2013, 2014; de Wild et al., 2013; Remy et al., 2013; Bouhlal et al., 2014). These findings further highlight the powerful role of taste exposure in isolation during this period.

FNL is assumed to be mediated by a decreasing state of hunger (Mobini et al., 2007); children are typically hungry at the start of feeding and less hungry or satiated at the end. In repeated exposure conditions children are often allowed to eat to satiety or close to satiety. As a consequence additional energy density may not increase the reward experienced, explaining the lack of advantage of FNL over repeated exposure.

A further factor that might explain the lack of benefit of FFL or FNL over repeated exposure relates to the number of exposures provided, which is typically 10 in the interventions that compare different exposure regimes. Given that 8–10 exposures are sufficient to induce full acceptance of a food at the start of complementary feeding (Maier et al., 2007), there is little scope for any additional benefit of FFL or FNL when this number of exposures is provided, and differences between treatments would not be expected. However, there are practical reasons for exploring whether, when fewer exposures are provided, FFL or FNL might be beneficial, as mothers are reluctant to provide as many as 10 exposures to a disliked food in practice (Maier et al., 2007). Future research might explore whether FFL increases acceptance of a novel or initially disliked food at the very first encounter, for example.

The literature relating to observational learning shows that, by the second year of life, infants pay attention to the foods eaten by their parents, peers and other role models; they are more willing to taste and accept into their diets the foods they have seen others eating (Harper and Sanders, 1975 and 16 other papers, see Table 2). However, questions remain about the types of role model infants are most likely to imitate in the early stages of developing food preferences, and whether the effectiveness of these changes with age. Moreover, research is needed to establish the extent to which infants' food preferences result directly from social learning vs. from the shared availability of specific foods within a household or cultural group. While a large number of studies demonstrate a relationship between the foods that a child observes being eaten as an infant or toddler and those that are an accepted component of their diets later in life, the nature of this relationship is complex. Is it the child's early observational experiences that cement a food as acceptable in the longer term, or does this require ongoing experiences of observing others eat the food at mealtimes? Given the stability of the young child's eating environment and companions, the effects of social learning are likely to be cumulative in shaping the child's diet over many years. Moreover, it is possible that such effects are due not to the child's modeling of their parents' or peers' food choices but to the child's repeated exposure to the same foods eaten by their family members. In the home environment, exposure and observation are inextricably linked; longitudinal studies that manipulate these factors independently would be required to establish which is responsible for the strong correlation between the child's early eating environment and eating preferences in adulthood.

Further work is also needed to establish how early experiences with foods impact on children's developing knowledge of food categories, and how this supports food choice and intake. The literature in this area is small, but the papers reviewed indicate that, by the third year, children are aware of the circumstances in which foods should be eaten and which foods should be eaten together (Rozin et al., 1986; Macario, 1991; Nguyen, 2007b). They also suggest that young children are hesitant to accept foods they are unable to recognize from previous encounters (Cashdan, 1998; Brown and Harris, 2012b). Aldridge et al. (2009) recently argued that the neophobia children display at 2 years of age may be related to their uncertainty about how to categorize unfamiliar foods. Children must make assumptions about new foods based on their similarity in appearance, name or category to familiar foods; as a result, the child's attitude toward a new food might reflect their past positive or negative experiences with similar foods. Pliner (2008) suggests that, rather than being unable to categorize unfamiliar foods, early experiences with unpalatable new foods lead children to establish a category of novel foods that includes characteristic qualities such as “tastes unpleasant” and “may be dangerous to eat,” setting up a strong bias to avoid new foods. Further research is required to test these hypotheses. Is consumption of a food increased if infants are supported in recognizing a food or if similarities between a new and a liked food are highlighted? Can a positive (rather than negative) category of novel foods be promoted from the outset by providing infants with a wide variety of tasty foods from the onset of weaning?

With the exception of studies that compare the effects of conditioning techniques with repeated exposure, the literature has largely ignored questions about the relative usefulness of different learning strategies, or how the effectiveness of these strategies changes with age. Table 6 provides an overview of the ages at which each learning mechanism has been shown to be employed by infants. With the exception of associative learning, each strategy has been shown to play a role in young children's food-related behavior within each of the defined age ranges from 4 to 36 months. Having said that, there are notable differences between what children are learning through each strategy at each age, which may reflect developmental changes in how children learn, as well as in what they are learning about (Snow and McGaha, 2003). It is important to note, however, that the gaps in Table 6 indicate an absence of evidence, rather than evidence of absence. Thus, Table 6 highlights that the 8- to 24-month age group has been relatively little explored in terms of how they learn about food; most studies have involved infants in the early weaning period or toddlers older than 2 years. Future work should explore whether these missing cells in the table are merely gaps in our knowledge, soon to be filled in, or genuine indications of sensitive periods, in which one type of learning takes precedence over others. We also know little about how children manage conflicting sources of evidence about foods—e.g., when they observe someone eating a novel food that looks like something they have categorized as disgusting, in the context of a liked odor. Do children attend to a single source of information in their response to the food—and if so, does observational, associative or categorical learning win out—or is their behavior based on a weighted combination of the different factors at play?

**Table 6:** Summary of what, how and when infants (weaning to 3 years) learn about food.

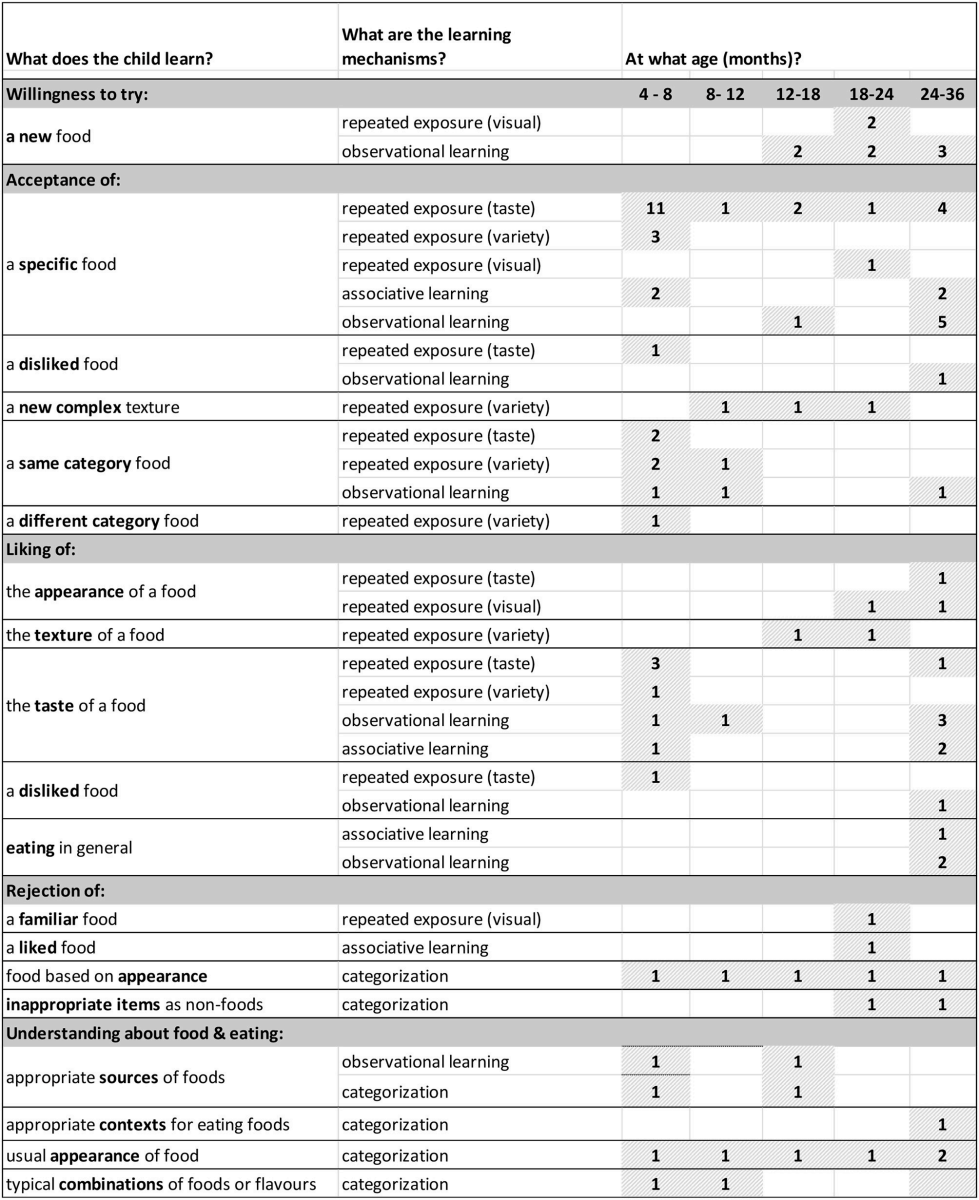

*Shaded areas in the table indicate that there is evidence in the literature that infants in the relevant age band use the specified learning strategy to learn about food. Blank cells indicate an absence of evidence in this age group. The number in each cell specifies the number of studies that have reported relevant evidence in this age group*.

We cannot be certain that the literature on which this review is based has uncovered all pertinent learning mechanisms. Goswami (2008a) identified four basic ways in which young children learn: (i) learning by imitation (analogous to our “observational learning” theme); (ii) learning by analogy (finding correspondences between events, situations or domains of knowledge in order to transfer knowledge from one to another), a formulation that overlaps with our conceptualization of “categorization”; (iii) statistical learning (detection of regularities in sensory input over time). Insofar as repeated exposure teaches that a food is safe to eat through the lack of aversive consequences following each encounter (Kalat and Rozin, 1973), familiarization constitutes a form of statistical learning. The learning of associations through flavor-flavor or FNL would similarly result from statistical learning over multiple encounters; and (iv) causal learning (the construction of causal explanations of the consequences of events). Gripshover and Markman (2013) report that 4-year olds reason causally about foods when learning about nutrition, but this form of learning has not been explored in children under 3 years, and is not considered by the literature reviewed here.

A further gap in the literature, aside from the question of “how” children learn about foods, is a comprehensive account of “what” they learn. Table 6 summarizes what we know children learn through each of the learning processes considered. The majority of studies have explored factors relating to children's evaluations of foods (whether a food is liked, preferred, accepted into their diet, or rejected). A smaller number of studies have investigated children's learning about the characteristics of foods. Other components of the knowledge children acquire about food remain unexplored. How do children learn the names of foods, the cultural and social contexts in which foods are eaten, foods' origins and preparation methods, and skills related to eating (e.g., how to use a spoon)? These questions are clearly also relevant to the development of healthy eating habits.

This review article has identified and summarized the literature on how infants learn about food between weaning and 36 months of age, and has confirmed that the learning strategies that support children's development in other domains are applicable to how children learn about food. The summary allows strong conclusions to be drawn in terms of the powerful role of familiarization with foods through repeated exposure; in our view, these can be communicated with confidence to parents and health workers who support and advise on the weaning process. The review has also highlighted gaps in our understanding of the operation of other learning strategies where the evidence is less clear-cut but clearly warrants further investigation. Given the well-documented relationship between children's early food preferences and food-related behaviors and dietary habits and health in later life, we would encourage a systematic interrogation of the questions this review has raised.

## Author contributions

MM conducted the systematic search and wrote the first version of the introduction, methodology section and part of the results section. CV and HW conceptualized the review. CV wrote part of the introduction, results and discussion. HW wrote part of the results and discussion. SC wrote part of the results and discussion. CH wrote part of the results section and discussion section. CH edited the full paper. All authors contributed to and approved the final manuscript.

### Conflict of interest statement

MM, CV, and HW are employees of Nutricia Research. The other authors declare that the research was conducted in the absence of any commercial or financial relationships that could be construed as a potential conflict of interest.
